# Supplementary material for: Safety outcomes when switching between biosimilars and reference biologics: A systematic review and meta-analysis
Source: PLoS One. 2023 Oct 3;18(10):e0292231. doi: 10.1371/journal.pone.0292231 (PMC10547155; doi:10.1371/journal.pone.0292231)
Supplement: S1 File — (DOCX) [file pone.0292231.s001.docx]

**Supporting information**

**Safety outcomes when switching between biosimilars and reference biologics**

**A systematic review and meta-analysis**

**Thomas M. Herndon, Cristina Ausin, Nina N. Brahme, Sarah J. Schrieber, Michelle Luo, Frances C. Andrada, Carol Kim, Wanjie Sun, Lingjie Zhou, Stella Grosser, Sarah Yim, M. Stacey Ricci**

**Safety outcomes when switching between biosimilars and reference biologics**

**A systematic review and meta-analysis**

**Supporting information**

**Table of Contents**

Additional material 3

Biosimilar development paradigm 4

Search strategy 5

Bias assessment 5

Heterogenicity analyses 5

Supplementary figures 6

Fig S1. Study schema for a typical safety and efficacy study included in systematic review 6

Fig S2. Switch patterns included in systematic review 7

Fig S3. Risk of death when switching: Adalimumab products .8

Fig S4. Risk of severe adverse events when switching: Adalimumab products 9

Fig S5. Risk of discontinuation when switching: Adalimumab products 10

Fig S6. Risk of death when switching: Infliximab products 11

Fig S7. Risk of severe adverse events when switching: Infliximab products 12

Fig S8. Risk of discontinuation when switching: Infliximab products 13

Fig S9. Risk of death when switching: Rituximab products 14

Fig S10. Risk of severe adverse events when switching: Rituximab products 15

Fig S11. Risk of discontinuation when switching: Rituximab products 16

Fig S12. Switching from reference biologic to biosimilar - risk difference for death 17

Fig S13. Switching from reference biologic to biosimilar - risk difference for severe adverse events 18

Fig S14. Switching from reference biologic to biosimilar - risk difference for discontinuation 19

Fig S15. Switching from biosimilar to reference biologic - risk difference for death 20

Fig S16. Switching from biosimilar to reference biologic - risk difference for severe adverse events 21

Fig S17. Switching from biosimilar to reference biologic - risk difference for discontinuation 22

Fig S18. Switching once from reference biologic to biosimilar - risk difference for death 23

Fig S19. Switching once from reference biologic to biosimilar - risk difference for severe adverse events 24

Fig S20. Switching once from reference biologic to biosimilar - risk difference for discontinuation 25

Fig S21. Switching once from reference biologic to biosimilar or biosimilar to reference biologic - risk difference for death 26

Fig S22 Switching once from reference biologic to biosimilar or biosimilar to reference biologic - risk difference for severe adverse events 27

Fig S23. Switching once from reference biologic to biosimilar or biosimilar to reference biologic - risk difference for discontinuation 28

Fig S24. Switching between reference biologic to biosimilar (multi-switch) - risk difference for death 29

Fig S25. Switching between reference biologic to biosimilar (multi-switch) - risk difference for severe adverse

events 30

Fig S26. Switching between reference biologic to biosimilar (multi-switch) - risk difference for discontinuation 31

Fig S27. Patients with antidrug antibody and neutralizing antibody positive assay results 32

Fig S28. Risk of bias 33

Supplementary tables 34

Table S1. Preferred Reporting Items for Systematic reviews and Meta-Analyses (PRISMA) checklist 34

Table S2. Additional information on included switch treatment periods 37

Table S3. Demographics (age, sex, body mass index) of patients for included switch treatment periods 42

Table S4. Ethnicity and race of patients for included switch treatment periods 47

Table S5. Exposure to study drug 54

Table S6. Deaths, severe adverse events, and discontinuations 57

Table S7. Antidrug antibody and neutralizing antibody data 59

Table S8. Hypersensitivity, infusion reactions, and injection site reactions 62

Table S9. Antidrug antibody and neutralizing antibody status in patients with hypersensitivity, infusion reactions, and injection site reactions 64

Table S10. Un-adjusted and adjusted odds ratio of death by individual factor and biosimilar drug 65

Table S11. Un-adjusted and adjusted odds ratio of SAE by individual factor and biosimilar drug 66

Table S12. Un-adjusted and adjusted odds ratio of discontinuation individual factor and biosimilar drug 67

References 68

**Additional material**

**Biosimilar development paradigm**

FDA approves reference products, biosimilar products (biosimilars), and interchangeable biosimilar products through different statutory approval pathways. A reference product is approved in a standalone 351(a) biologics license application (BLA), which must contain all data and information necessary to demonstrate the product’s safety and effectiveness and generally includes data from clinical trials conducted in the relevant patient populations for each of the treatment indications being sought by the manufacturer. A biosimilar, by definition, is highly similar to and has no clinically meaningful differences in terms of safety, purity, and potency (i.e., safety and effectiveness) from an FDA-approved reference product. The abbreviated approval pathway for biosimilars was created to help reduce the time and cost of development of biologics without compromising safety and effectiveness. The goal of a biosimilar development program is to demonstrate biosimilarity between the proposed biosimilar and its reference product, not to independently establish the safety and effectiveness of the biosimilar. Therefore, all biosimilar and interchangeable biosimilar products are approved through the abbreviated 351(k) pathway, which is based on a comparison of the biosimilar to the reference product (1).

The biosimilar manufacturer generates an array of data comparing the proposed biosimilar to the FDA-approved reference product to demonstrate biosimilarity. They do not have to generate the same package of nonclinical and clinical data as required for the reference product. Rather, the biosimilar manufacturer provides comparative data beginning with a detailed analytical (structural and functional) comparison of the reference product and proposed biosimilar. Manufacturers also conduct clinical studies comparing the proposed biosimilar to the reference biologic, which typically include PK, PD/efficacy, safety and immunogenicity evaluations. The totality of these comparative data supports FDA’s determination whether the proposed biosimilar is highly similar to and has no clinically meaningful differences from an FDA-approved reference product.

**Analytics**

Comparative analytical data provide the foundation for the development of a proposed biosimilar for submission in an application submitted to FDA. Advances in manufacturing science and production methods have enabled the extensive characterization necessary to show that a proposed biosimilar can be demonstrated to be analytically highly similar to its reference product. Biosimilar manufacturers accomplish this by first determining the quality attributes that characterize the reference product in terms of the physicochemical and functional properties that impact PK, PD, safety, efficacy, and/or immunogenicity and designing a manufacturing process to produce a product with these quality attributes. The comparative analytical assessment verifies that each of these quality attributes, as observed in the proposed biosimilar and the reference product, are similar using quantitative and/or qualitative analyses. Physicochemical properties assessed include but are not limited to comparisons of amino acid sequence, higher order structure related to protein folding and sub-unit assembly (if relevant), the types and amounts of glycan structures and other post-translational modifications, and characterizing variants in terms of size and charge. The functional properties assessed reflect the clinically relevant mechanism(s) of action for the reference product to the extent possible, as well as other biological activities that can impact PK, PD/efficacy, immunogenicity and safety. The types of functional tests used include in vitro cell-based assays with functional readouts (e.g., proliferation, cell death, gene expression), ligand or receptor binding affinity, enzymatic activity, etc. The analytical testing methods used for these comparisons are generally much more sensitive for identifying differences between a proposed biosimilar and its reference product than the results of a clinical study.

Additional information can be found on FDA’s website(1) and guidance publications (2, 3).

**Comparative Clinical Study**

Comparative clinical studies included in this review had a study design similar to the general study schema shown in Fig S1. All the biosimilars reviewed in our submission are FDA-approved products. For each biosimilar, there was at least one clinical study powered for efficacy (or PD) using endpoints that were clinically relevant to the population studied included in the BLA submission and reviewed by FDA. A typical clinical study design for the studies included in our review has two or more treatment periods. Comparisons of efficacy are evaluated during Treatment Period 1 which is powered to establish that the proposed biosimilar is neither inferior nor superior to the reference product. After the primary endpoint is collected, patients enter Treatment Period 2 which contains one or more switches between the reference product and the proposed biosimilar product. While some efficacy data may be collected during Treatment Period 2, this portion of the study during the switch is not powered for efficacy comparisons, as the primary concern following switching from the reference biologic to the proposed biosimilar is whether there is a major risk in terms of hypersensitivity, immunogenicity, or other reaction. In addition, the clinical efficacy measurements after the primary endpoint would generally be less sensitive for detecting changes in exposure and/or activity after switching. After the completion of Treatment Period 2, patients may be assigned to Treatment Period 3, an extension period where all patients remaining are given the biosimilar product. Treatment Period 3 contains a switch for the patients who were on the reference product arm during Treatment Period 2.

**Search strategy**

Searches were performed for the period of 01 January 2000 through 31 December 2022. FDA databases containing publicly available information were reviewed for all clinical studies submitted as part of a BLA for an approved biosimilar. The Embase database was queried with the assistance of a reference librarian using the following search syntax: 'biosimilar agent'/exp OR 'biosimilar agent' OR 'biosimilar drug'/exp OR 'biosimilar drug' OR biosimilar* OR 'biologic factors' AND 'drug substitution'/exp OR 'drug substitution' OR 'switching'/exp OR switching OR 'transition'/exp OR transition. Through Embase, the MEDLINE data base was queried using the same search terms. PubMed was searched for recent publications that may not have been indexed into Embase or Medline. No language restrictions were applied. Non-human studies, and any publication that was not classified by Embase as an “Article”, “Article in Press”, or “Review” were excluded.

**Bias assessment**

Two reviewers independently assessed risk of bias for each STP using a modified version of the Newcastle-Ottawa Assessment Scale (mNOAS) (4). As our review consists of STPs that may not have been from the randomized portion of the larger study, the mNOAS was chosen as it has been used by others to address subgroups within a randomized trial as independent observational cohorts.(5, 6) Using the mNOAS allowed for comparisons of bias across all STPs. STPs that scored 4 or more on the modified mNOAS were considered to have a low risk of bias (5).

**Heterogenicity analyses**

Additional exploratory logistic regression analyses were performed to evaluate the impact of heterogeneity of different study design and patient characteristics on study conclusions. Adjusted Odds Ratios (OR) from multiple logistic regression models were adjusted for the biosimilar drugs assessed and each individual factor (study design, patient population, time on study drug prior to switching, duration of switch, number of switches during STP, mean age, percent sex, and mean BMI). Due to the limitation of individual data for clinical studies, summary data based on aggregate information were used to conduct the exploratory Logistic regression, which may not be as accurate or efficient as when individual data is used.

**Supplementary figures**

Fig S1. Study schema for a typical safety and efficacy study included in systematic review.


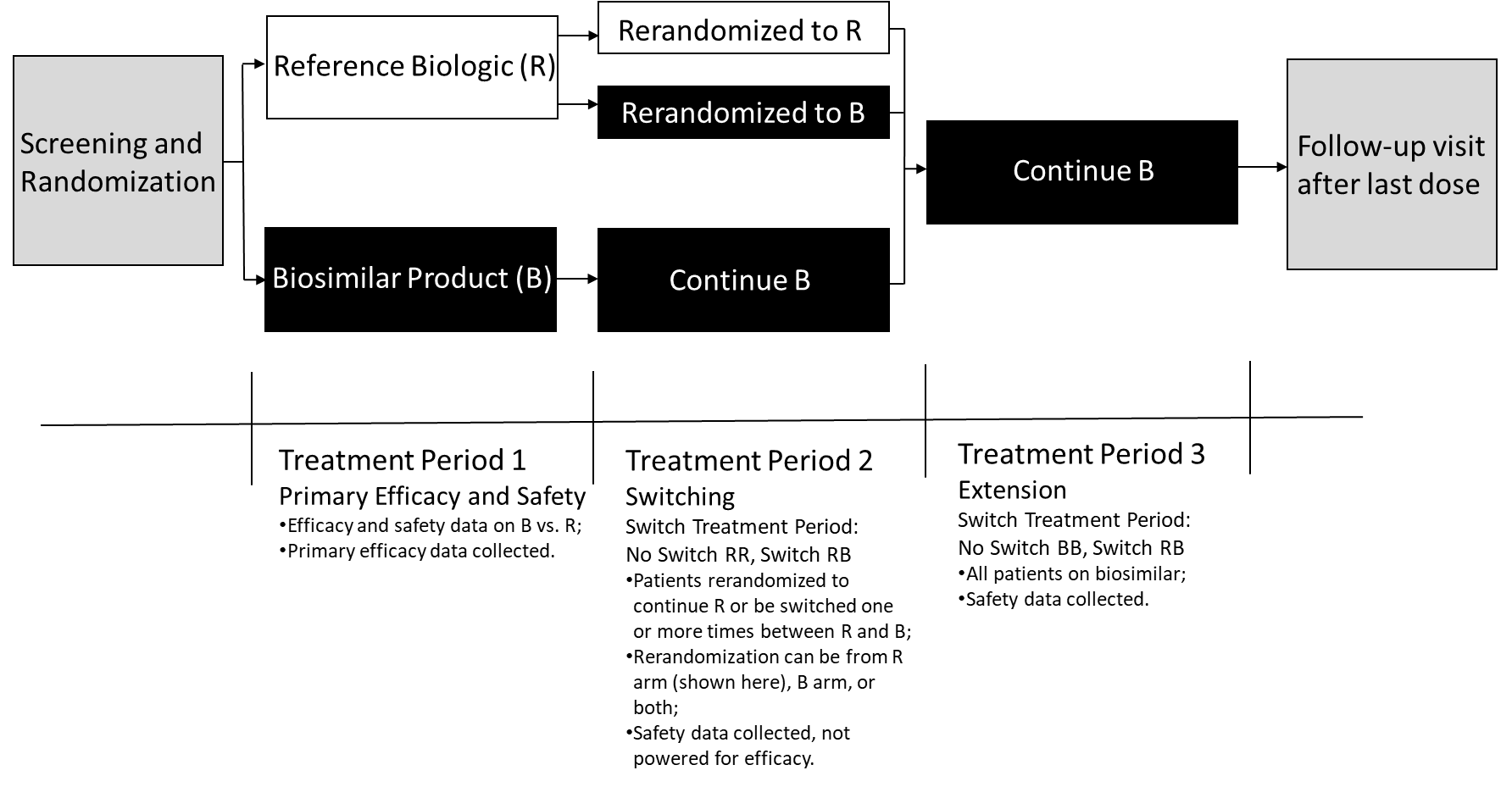


**Fig S2. Switch patterns included in systematic review.**

(B-B, B-R) is biosimilar to biosimilar in No Switch arm and biosimilar to reference biologic in Switch arm, (B-B, R-B) is biosimilar to biosimilar in No Switch arm and biosimilar to reference biologic in Switch arm. (R-R, R-B) is reference biologic to reference biologic in No Switch arm and reference biologic to biosimilar in Switch arm. STP is switch treatment period.


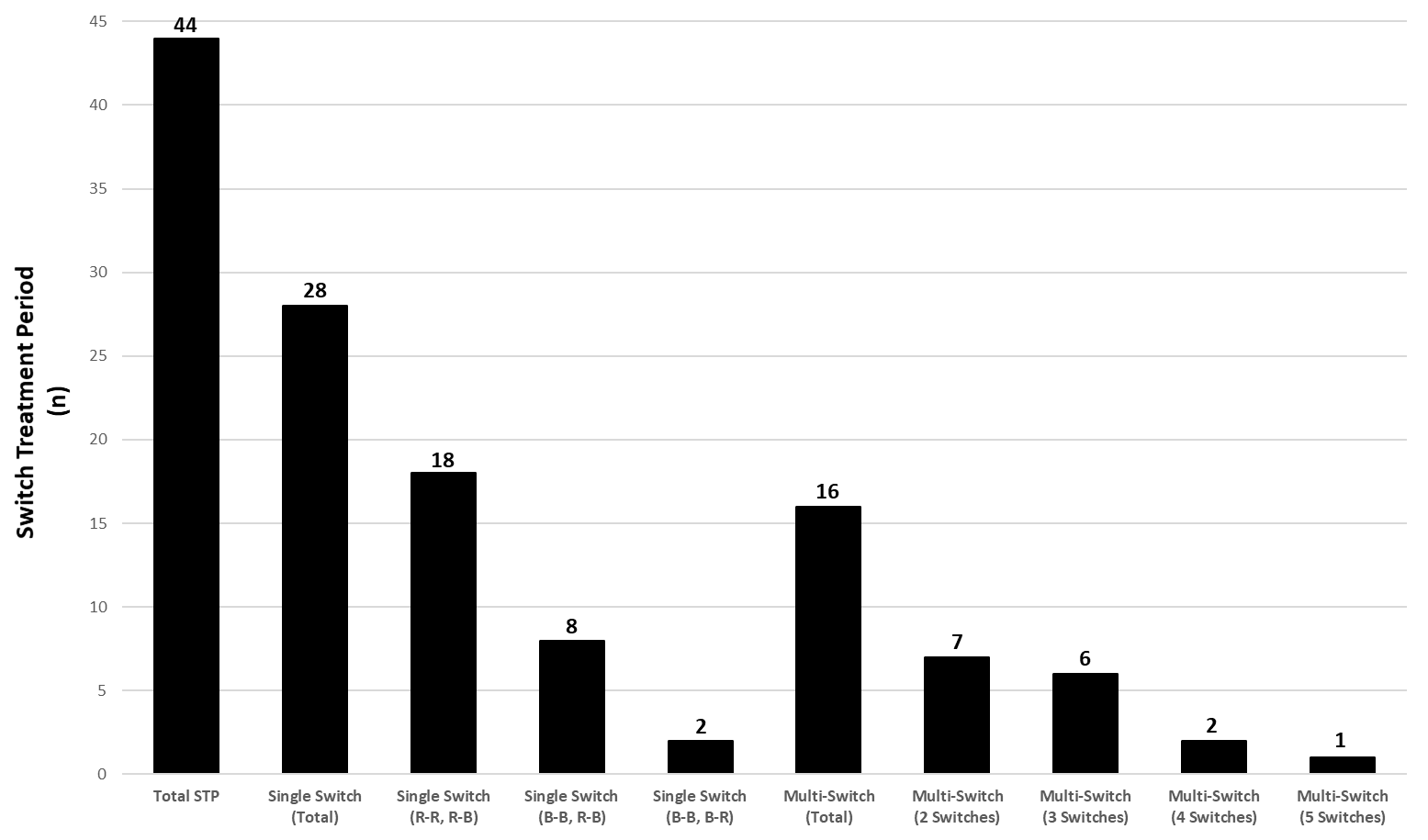


**Fig S3. Risk of death when switching: Adalimumab products.**

Meta-analysis was performed of the risk difference for death between Switch and No Switch in each switch treatment period (STP) containing a biosimilar to adalimumab. Weight refers to the contribution of each STP to the overall estimate of risk difference, which is based on the inverse of the variance of the respective risk difference. 𝜒^2 and df are used in the chi-square test for homogeneity of risk difference across studies. Z value is used in the normal Z test for whether the overall risk difference is zero. CI is the confidence interval.


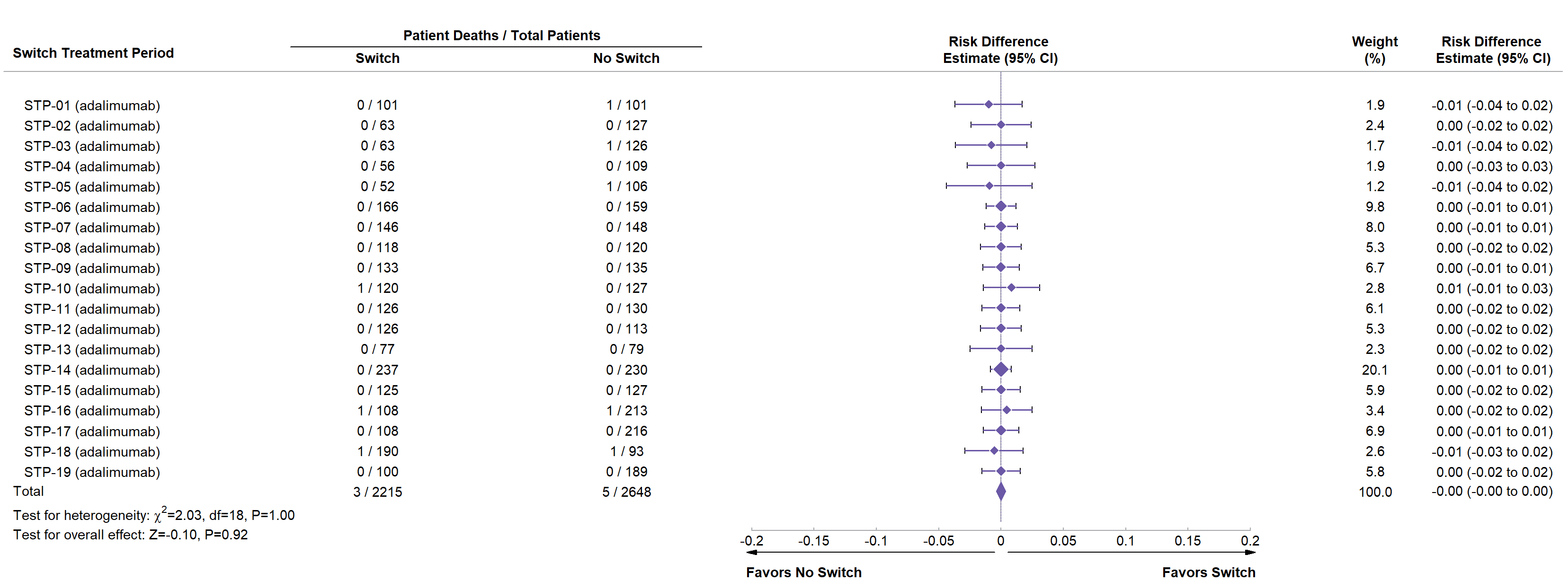


**Fig S4. Risk of severe adverse events when switching: Adalimumab products.**

Meta-analysis was performed of the risk difference for one or more serious adverse events between Switch and No Switch arms in each switch treatment period (STP) containing a biosimilar to adalimumab. Weight refers to the contribution of each STP to the overall estimate of risk difference, which is based on the inverse of the variance of the respective risk difference. 𝜒^2 and df are used in the chi-square test for homogeneity of risk difference across studies. Z value is used in the normal Z test for whether the overall risk difference is zero. CI is the confidence interval.


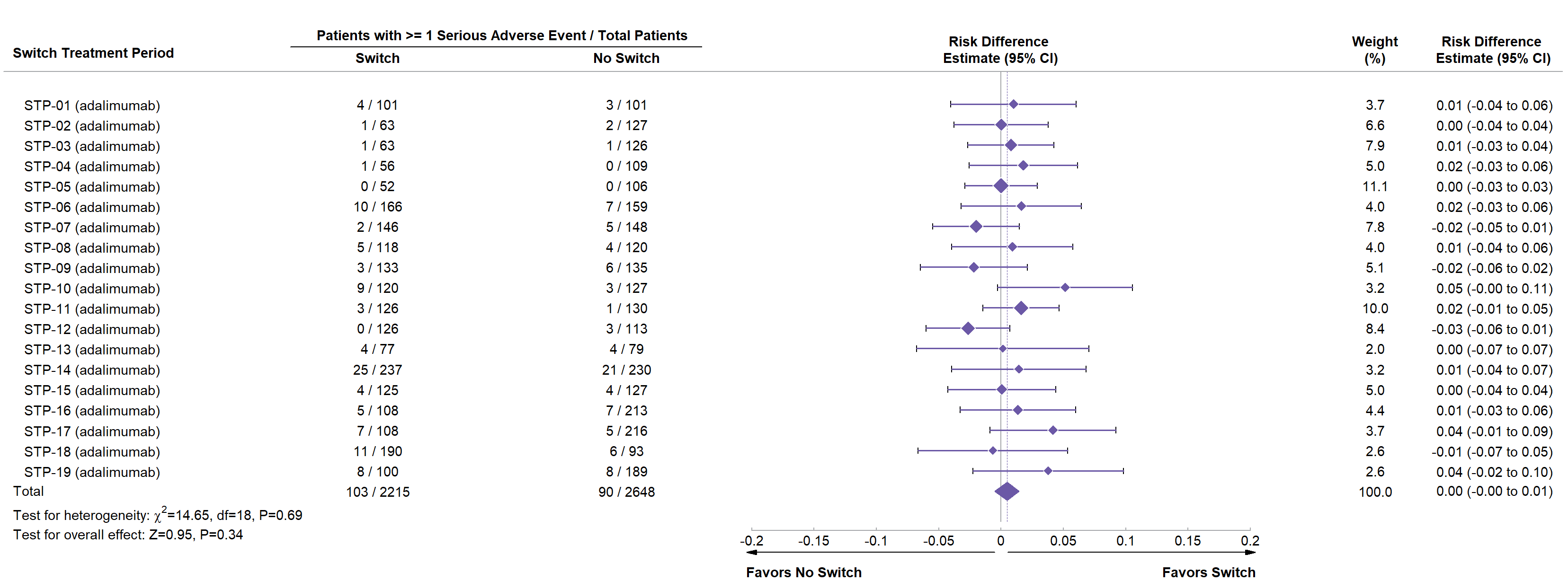


**Fig S5. Risk of discontinuation when switching: Adalimumab products.**

Meta-analysis was performed of the risk difference for permanent discontinuation of study drug due to an adverse event between Switch and No Switch arms in each switch treatment period (STP) containing a biosimilar to adalimumab. Weight refers to the contribution of each STP to the overall estimate of risk difference, which is based on the inverse of the variance of the respective risk difference. 𝜒^2 and df are used in the chi-square test for homogeneity of risk difference across studies. Z value is used in the normal Z test for whether the overall risk difference is zero. CI is the confidence interval.


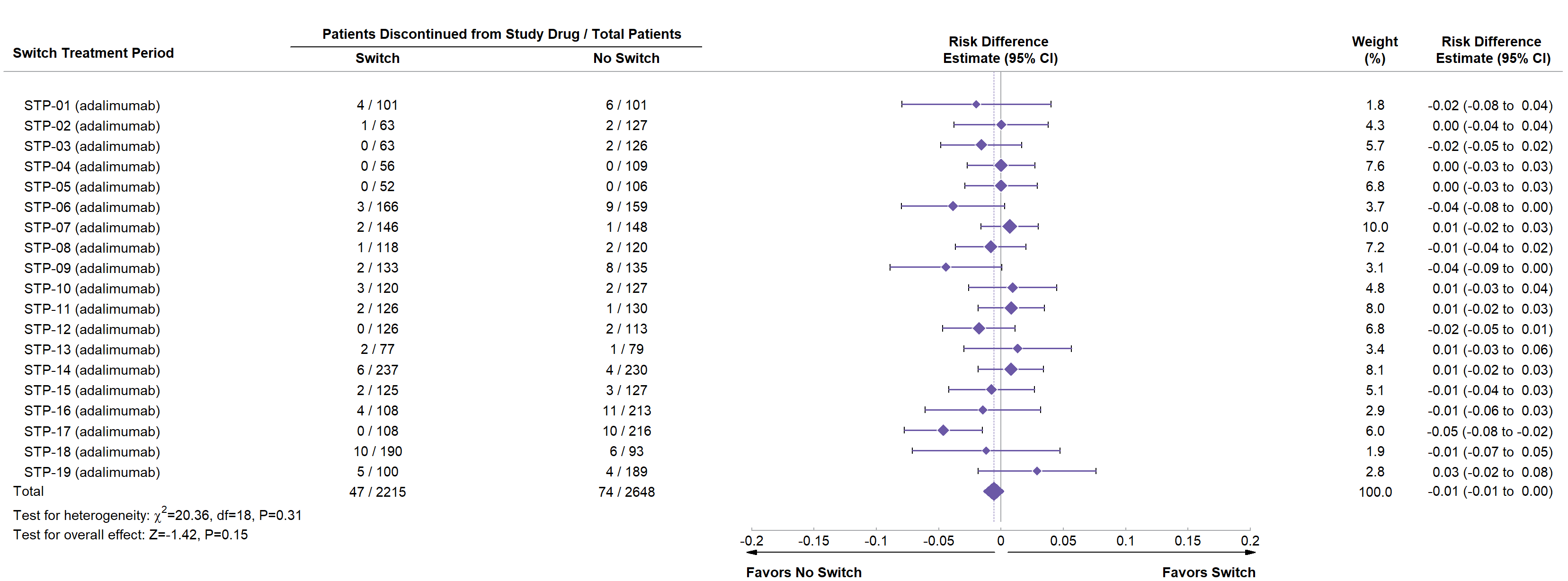


**Fig S6. Risk of death when switching: Infliximab products.**

Meta-analysis was performed of the risk difference for death between Switch and No Switch arms in each switch treatment period (STP) containing a biosimilar to infliximab. Weight refers to the contribution of each STP to the overall estimate of risk difference, which is based on the inverse of the variance of the respective risk difference. 𝜒^2 and df are used in the chi-square test for homogeneity of risk difference across studies. Z value is used in the normal Z test for whether the overall risk difference is zero. CI is the confidence interval.


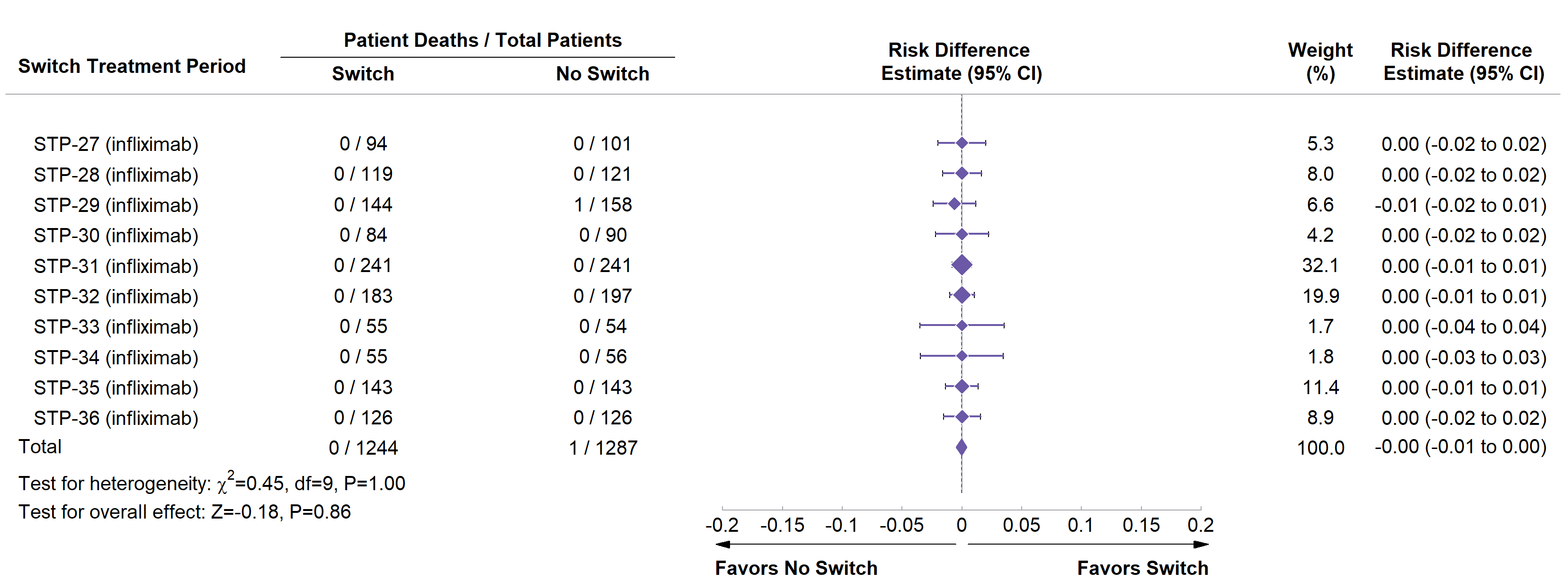


**Fig S7. Risk of severe adverse events when switching: Infliximab products.**

Meta-analysis was performed of the risk difference for one or more serious adverse events between Switch and No Switch arms in each switch treatment period (STP) containing a biosimilar to infliximab. Weight refers to the contribution of each STP to the overall estimate of risk difference, which is based on the inverse of the variance of the respective risk difference. 𝜒^2 and df are used in the chi-square test for homogeneity of risk difference across studies. Z value is used in the normal Z test for whether the overall risk difference is zero. CI is the confidence interval.


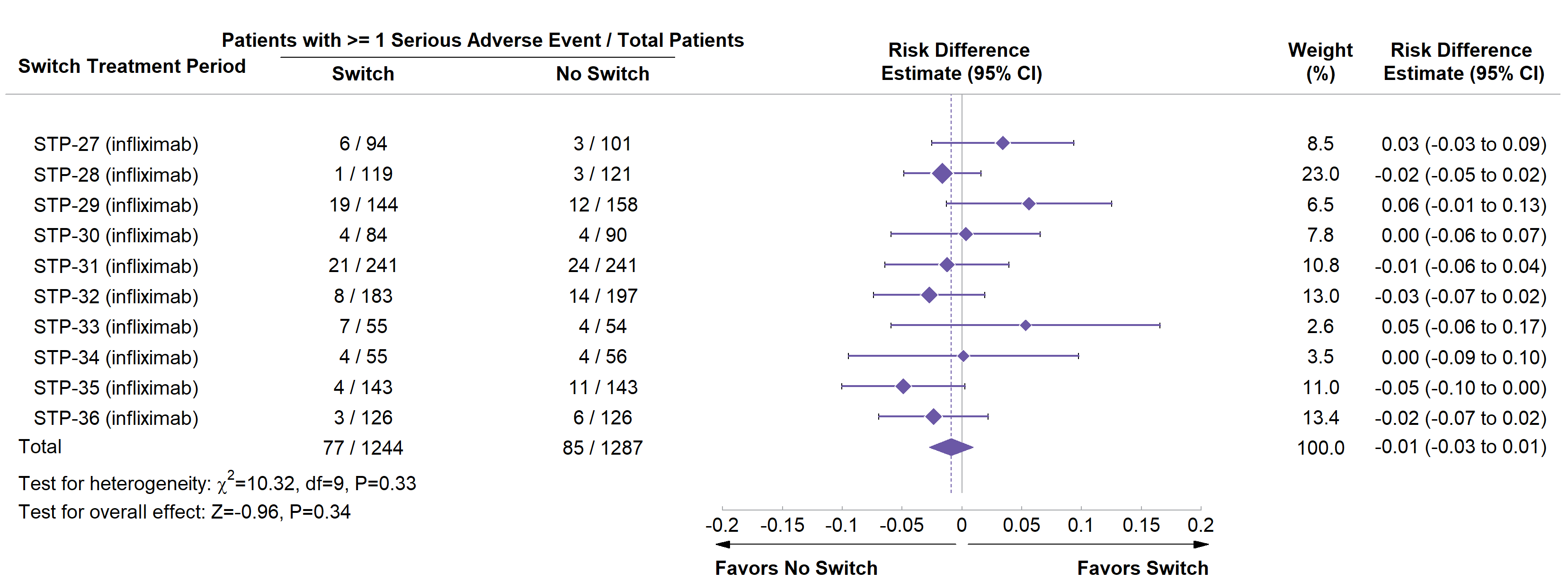


**Fig S8. Risk of discontinuation when switching: Infliximab products.**

Meta-analysis was performed of the risk difference for permanent discontinuation of study drug due to an adverse event between Switch and No Switch arm in each switch treatment period (STP) containing a biosimilar to infliximab. Weight refers to the contribution of each STP to the overall estimate of risk difference, which is based on the inverse of the variance of the respective risk difference. 𝜒^2 and df are used in the chi-square test for homogeneity of risk difference across studies. Z value is used in the normal Z test for whether the overall risk difference is zero. CI is the confidence interval.


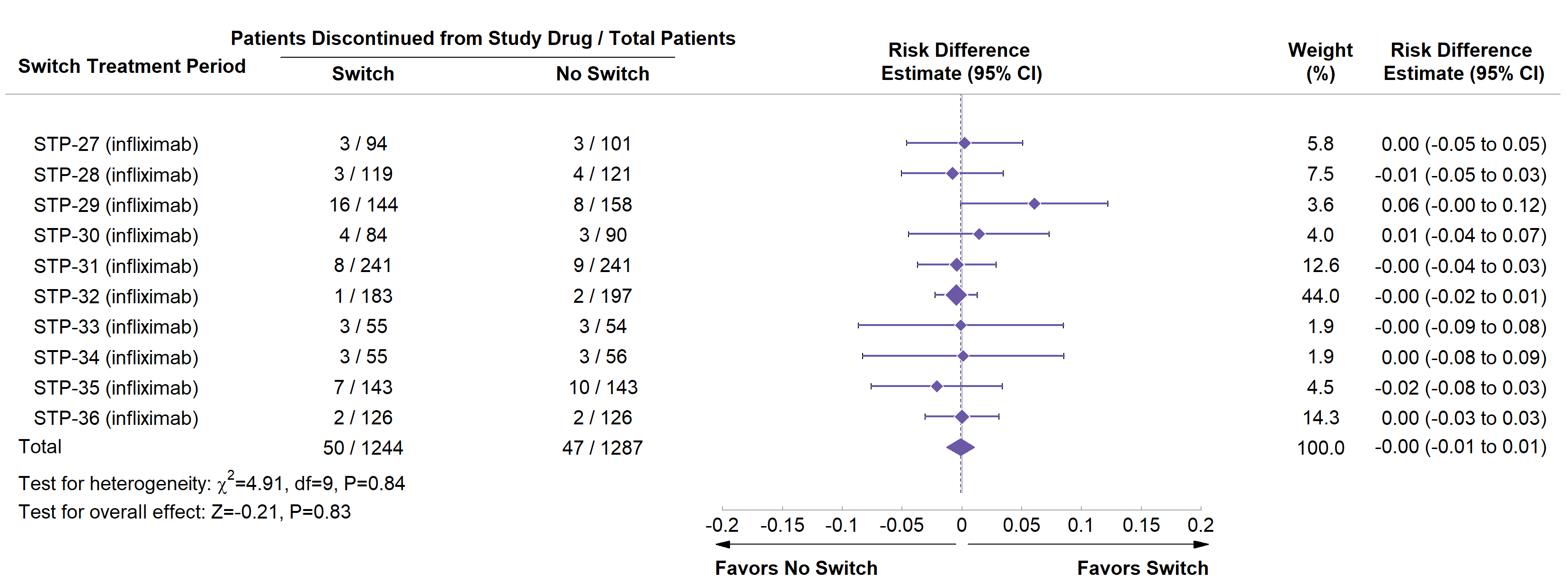


**Fig S9. Risk of death when switching: Rituximab products.**

Meta-analysis was performed of the risk difference for death between Switch and No Switch arms in each switch treatment period (STP) containing a biosimilar to rituximab. Weight refers to the contribution of each STP to the overall estimate of risk difference, which is based on the inverse of the variance of the respective risk difference. 𝜒^2 and df are used in the chi-square test for homogeneity of risk difference across studies. Z value is used in the normal Z test for whether the overall risk difference is zero. CI is the confidence interval.


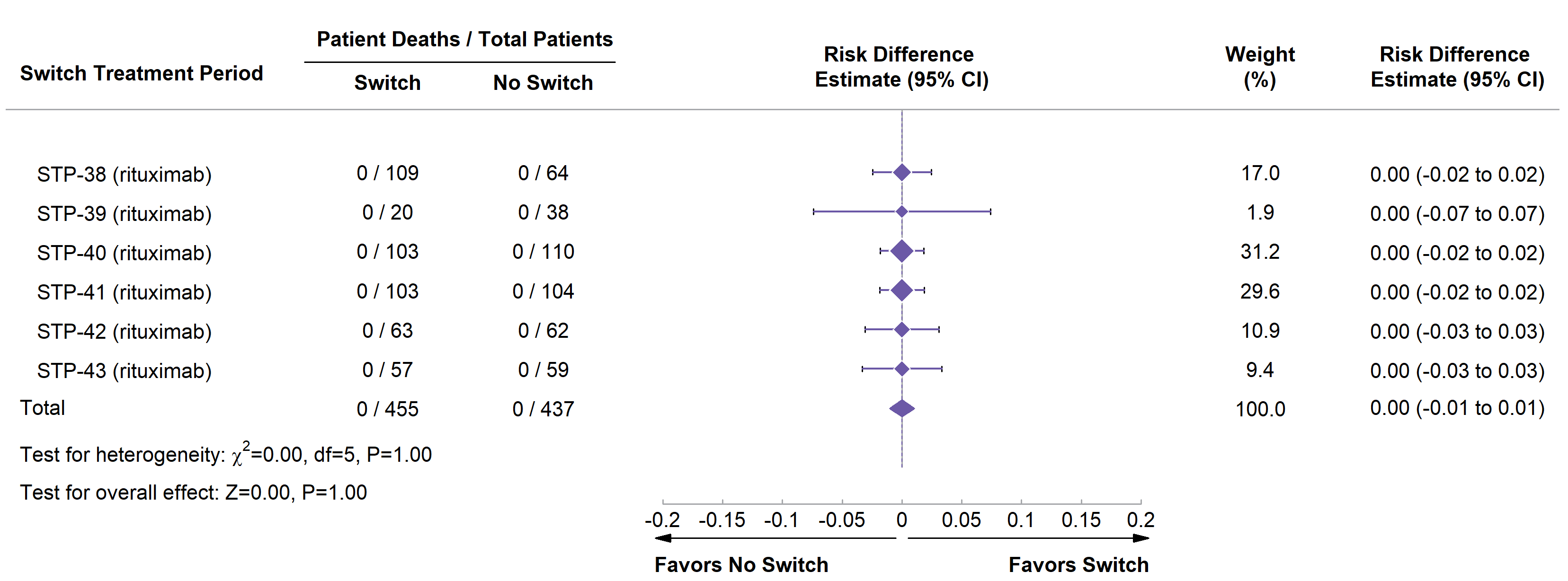


**Fig S10. Risk of severe adverse events when switching: Rituximab products.**

Meta-analysis was performed of the risk difference for one or more serious adverse events between Switch and No Switch arms in each switch treatment period (STP) containing a biosimilar to rituximab. Weight refers to the contribution of each STP to the overall estimate of risk difference, which is based on the inverse of the variance of the respective risk difference. 𝜒^2 and df are used in the chi-square test for homogeneity of risk difference across studies. Z value is used in the normal Z test for whether the overall risk difference is zero. CI is the confidence interval.


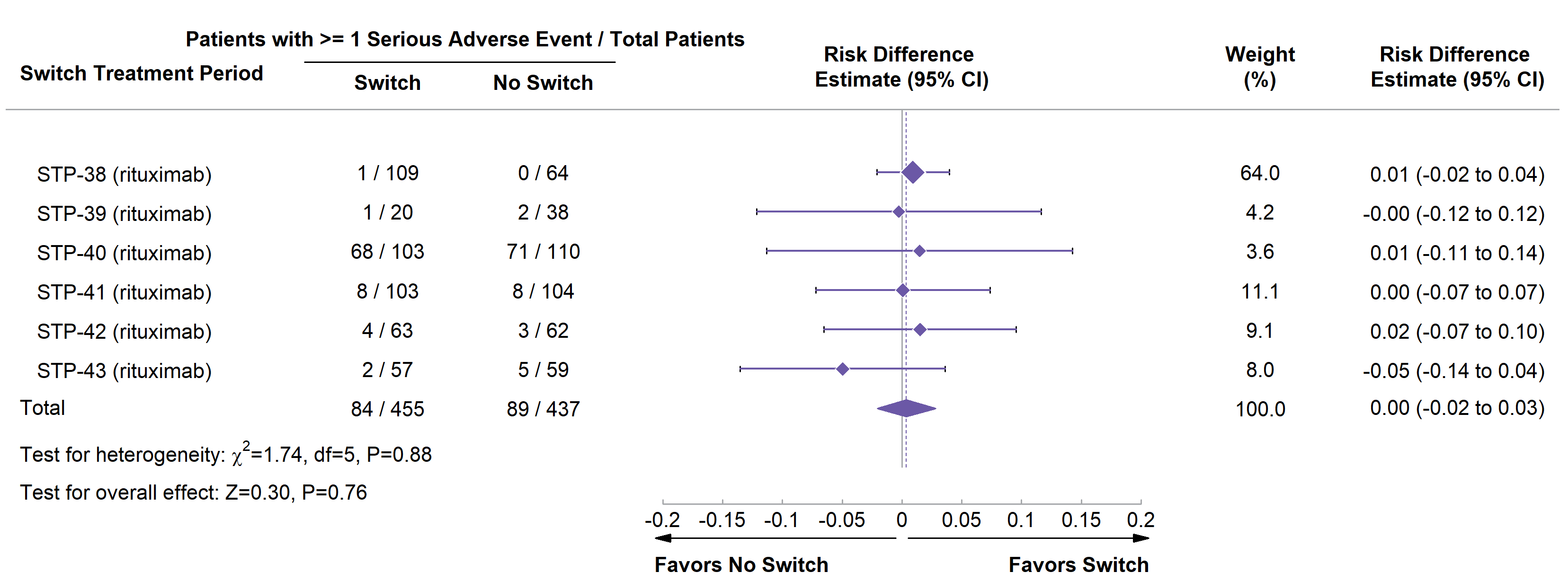


**Fig S11. Risk of discontinuation when switching: Rituximab products.**

Meta-analysis was performed of the risk difference for permanent discontinuation of study drug due to an adverse event between Switch and No Switch arms in each switch treatment period (STP) containing a biosimilar to rituximab. Weight refers to the contribution of each STP to the overall estimate of risk difference, which is based on the inverse of the variance of the respective risk difference. 𝜒^2 and df are used in the chi-square test for homogeneity of risk difference across studies. Z value is used in the normal Z test for whether the overall risk difference is zero. CI is the confidence interval.


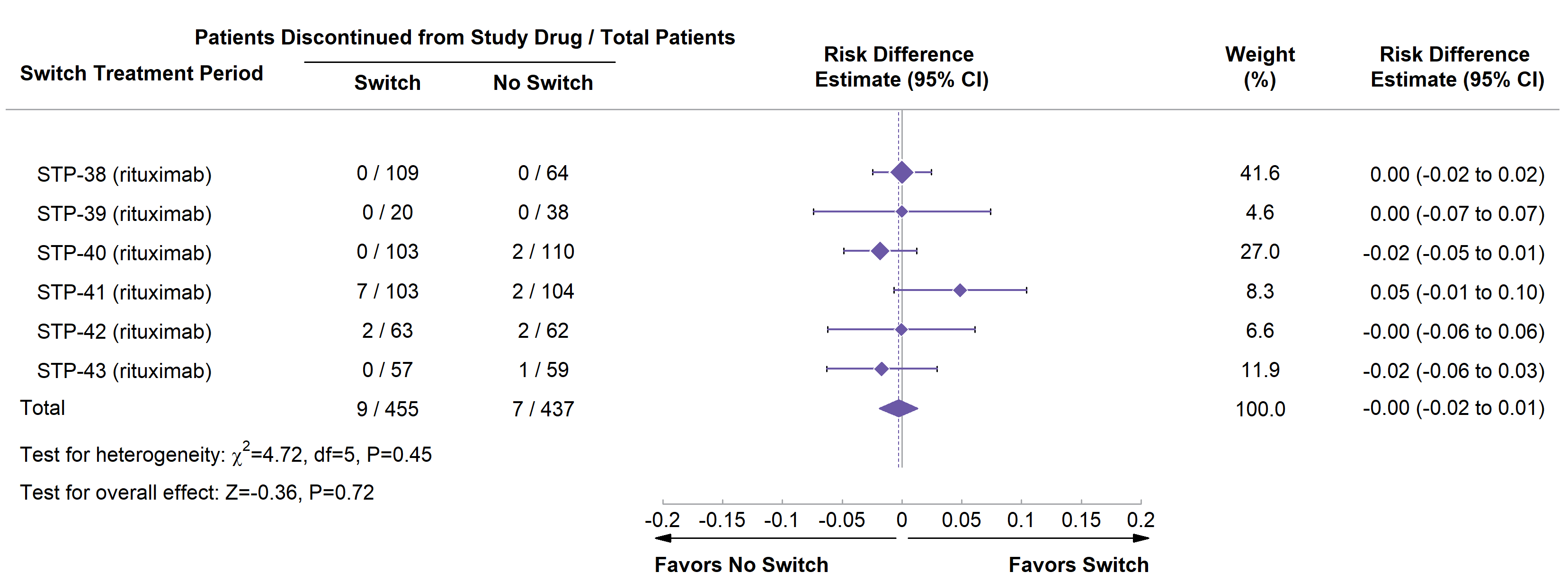


**Fig S12. Switching from reference biologic to biosimilar - risk difference for death.**

Meta-analysis was performed of the risk difference for death between Switch and No Switch arms in each switch treatment period (STP) containing a Switch arm where patients were switched one or more times between a reference biologic and a biosimilar (with the final switch being from reference biologic to biosimilar) and a No Switch arm where patients remained on the reference biologic. Weight refers to the contribution of each STP to the overall estimate of risk difference, which is based on the inverse of the variance of the respective risk difference. 𝜒^2 and df are used in the chi-square test for homogeneity of risk difference across studies. Z value is used in the normal Z test for whether the overall risk difference is zero. CI is the confidence interval.


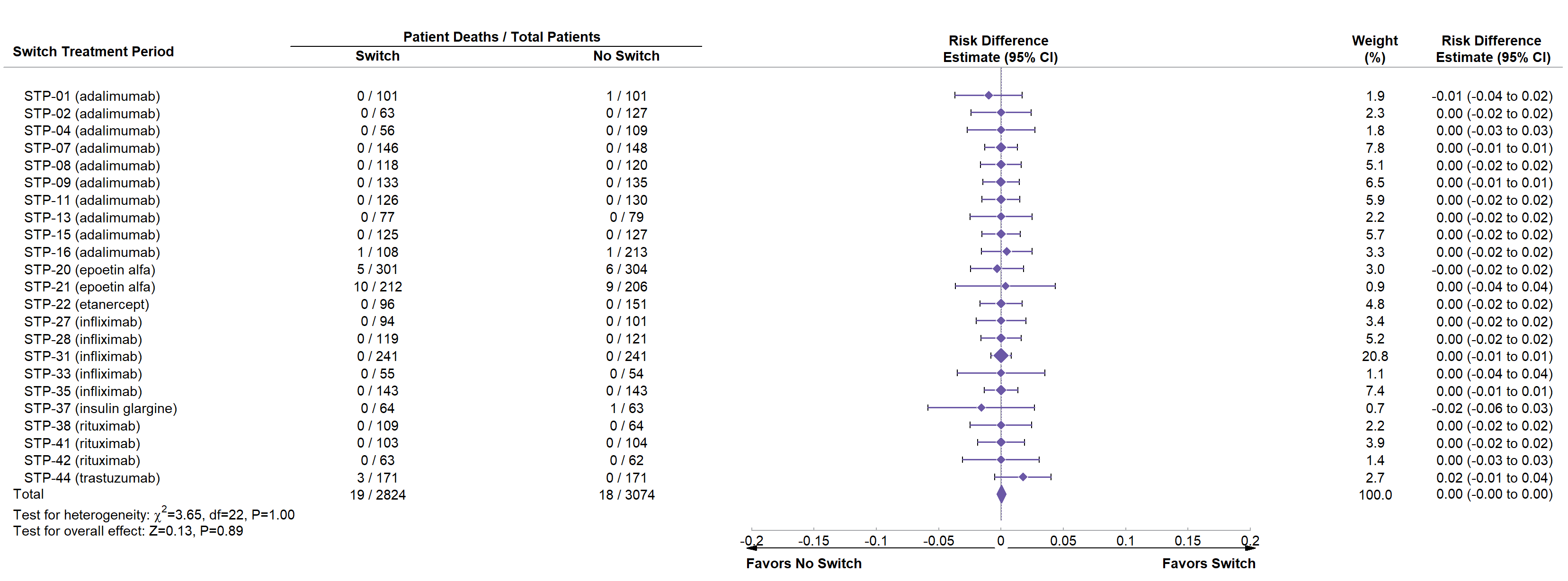


**Fig S13. Switching from reference biologic to biosimilar - risk difference for severe adverse events.**

Meta-analysis was performed of the risk difference for one or more serious adverse events between Switch and No Switch arms in each switch treatment period (STP) containing a Switch arm where patients were switched one or more times between a reference biologic and a biosimilar (with the final switch being from reference biologic to biosimilar) and a No Switch arm where patients remained on the reference biologic. Weight refers to the contribution of each STP to the overall estimate of risk difference, which is based on the inverse of the variance of the respective risk difference. 𝜒^2 and df are used in the chi-square test for homogeneity of risk difference across studies. Z value is used in the normal Z test for whether the overall risk difference is zero. CI is the confidence interval.


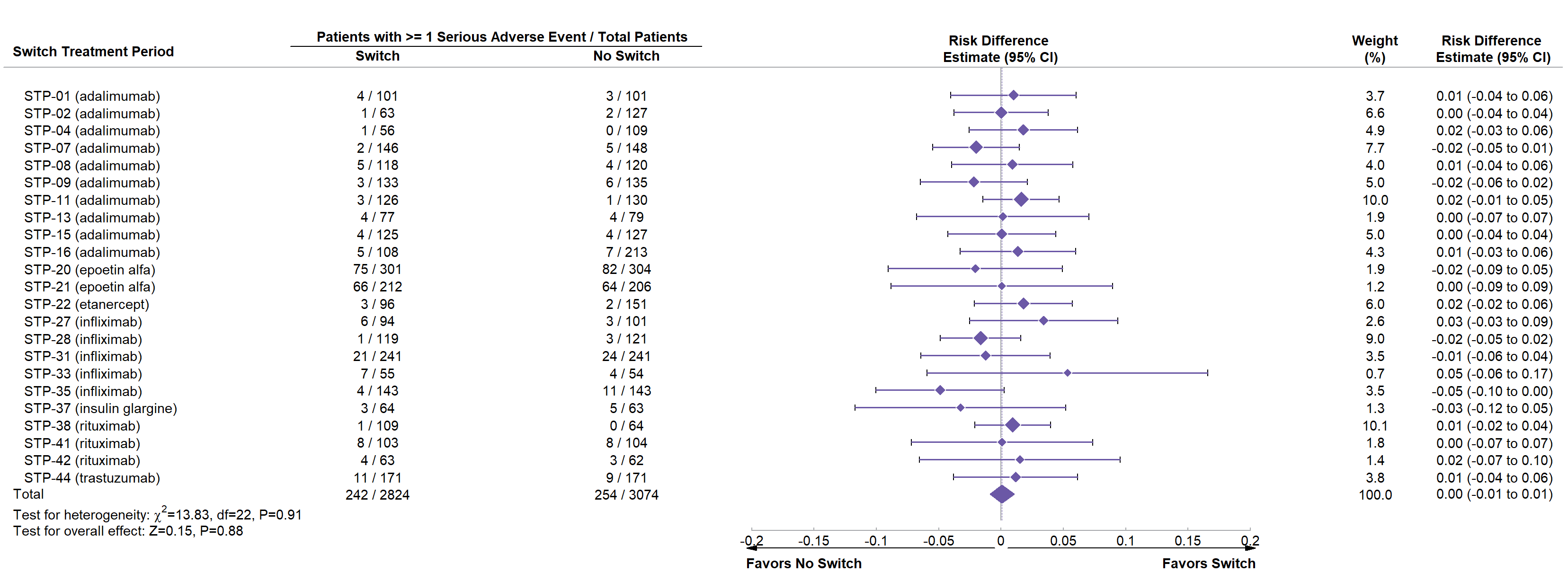


**Fig S14. Switching from reference biologic to biosimilar - risk difference for discontinuation.**

Meta-analysis was performed of the risk difference for permanent discontinuation of study drug due to an adverse event between Switch and No Switch in each switch treatment period (STP) containing a Switch arm where patients were switched one or more times between a reference biologic and a biosimilar (with the final switch being from reference biologic to biosimilar) and a No Switch arm where patients remained on the reference biologic. Weight refers to the contribution of each STP to the overall estimate of risk difference, which is based on the inverse of the variance of the respective risk difference. 𝜒^2 and df are used in the chi-square test for homogeneity of risk difference across studies. Z value is used in the normal Z test for whether the overall risk difference is zero. CI is the confidence interval.


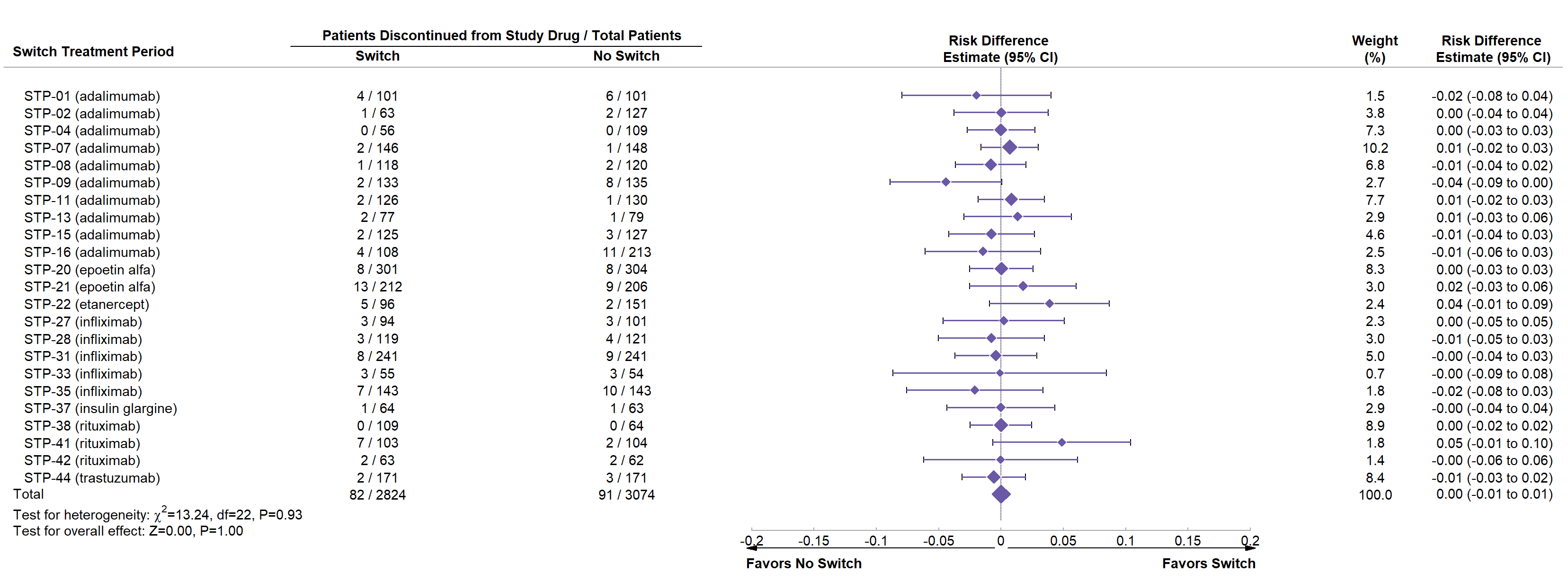


**Fig S15. Switching from biosimilar to reference biologic - risk difference for death.**

Meta-analysis was performed of the risk difference for death between Switch and No Switch arms in each switch treatment period (STP) containing a Switch arm where patients were switched one or more times between a reference biologic and a biosimilar (with the final switch being from biosimilar to reference biologic) and a No Switch arm where patients remained on the biosimilar. Weight refers to the contribution of each STP to the overall estimate of risk difference, which is based on the inverse of the variance of the respective risk difference. 𝜒^2 and df are used in the chi-square test for homogeneity of risk difference across studies. Z value is used in the normal Z test for whether the overall risk difference is zero. CI is the confidence interval.


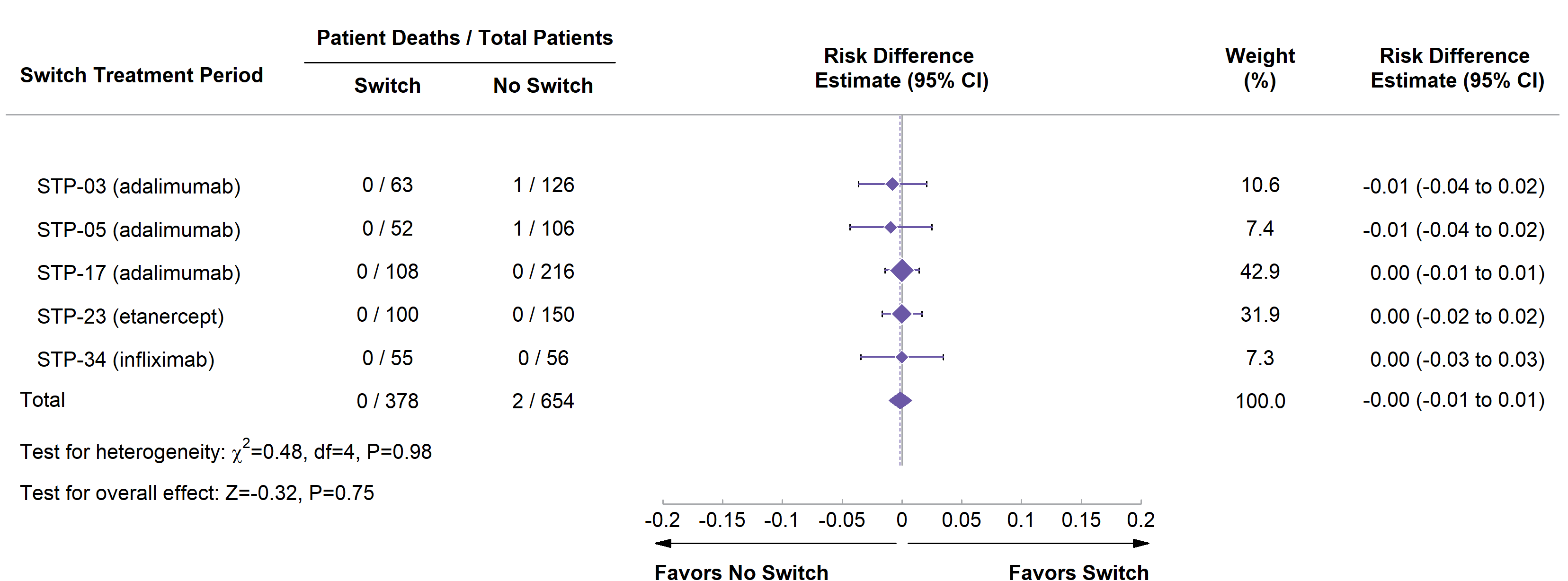


**Fig S16.** **Switching from biosimilar to reference biologic - risk difference for severe adverse events.**

Meta-analysis was performed of the risk difference for one or more serious adverse events between Switch and No Switch arms in each switch treatment period (STP) containing a Switch arm where patients were switched one or more times between a reference biologic and a biosimilar (with the final switch being from biosimilar to reference biologic) and a No Switch arm where patients remained on the biosimilar. Weight refers to the contribution of each STP to the overall estimate of risk difference, which is based on the inverse of the variance of the respective risk difference. 𝜒^2 and df are used in the chi-square test for homogeneity of risk difference across studies. Z value is used in the normal Z test for whether the overall risk difference is zero. CI is the confidence interval.


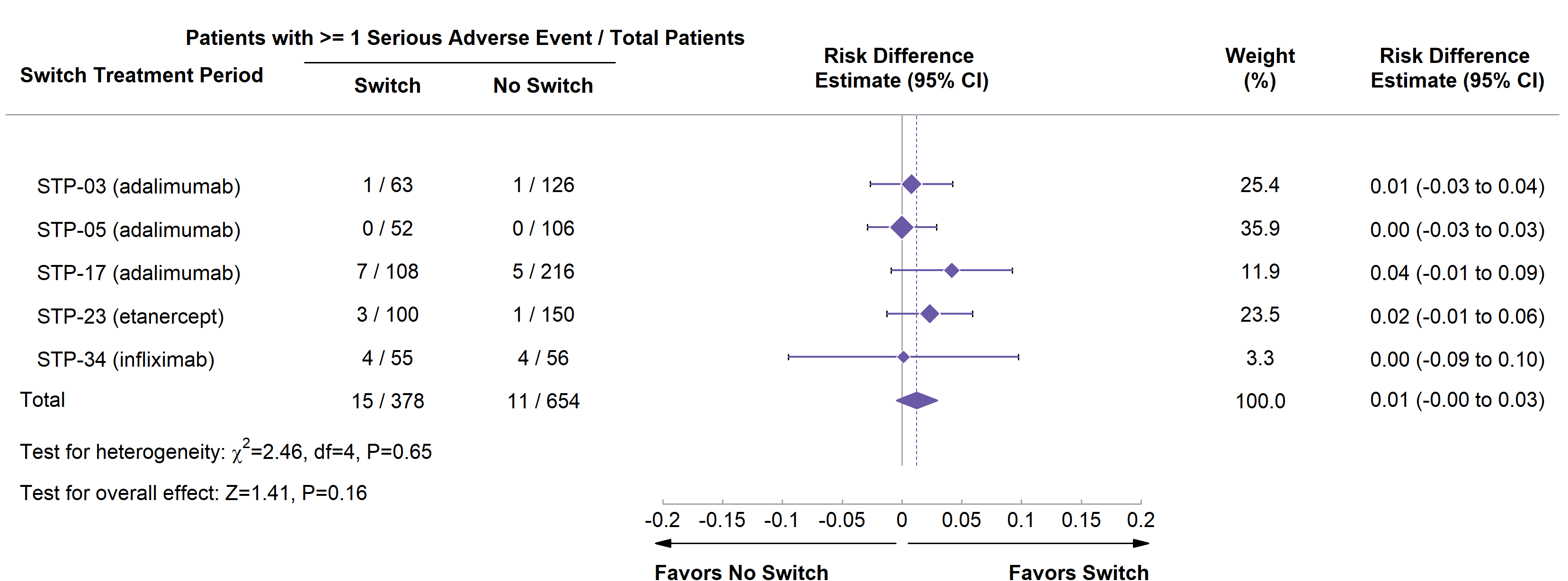


**Fig S17. Switching from biosimilar to reference biologic - risk difference for discontinuation.**

Meta-analysis was performed of the risk difference for permanent discontinuation of study drug due to an adverse event between Switch and No Switch in each switch treatment period (STP) containing a Switch arm where patients were switched one or more times between a reference biologic and a biosimilar (with the final switch being from biosimilar to reference biologic) and a No Switch arm where patients remained on the biosimilar. Weight refers to the contribution of each STP to the overall estimate of risk difference, which is based on the inverse of the variance of the respective risk difference. 𝜒^2 and df are used in the chi-square test for homogeneity of risk difference across studies. Z value is used in the normal Z test for whether the overall risk difference is zero. CI is the confidence interval.


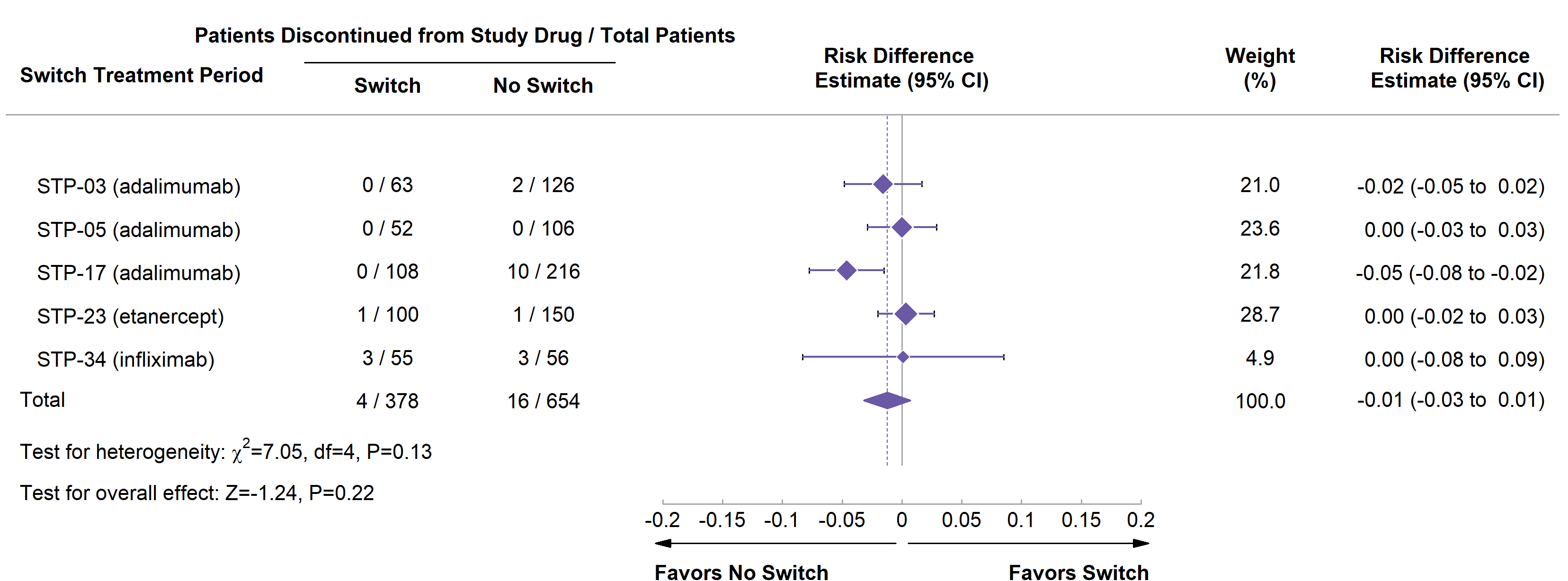


**Fig S18. Switching once from reference biologic to biosimilar - risk difference for death.**

Meta-analysis was performed of the risk difference for death between Switch and No Switch arms in each switch treatment period (STP) containing a Switch arm where patients were switched once from a reference biologic to a biosimilar and a No Switch arm where patients remained on the reference biologic. Weight refers to the contribution of each STP to the overall estimate of risk difference, which is based on the inverse of the variance of the respective risk difference. 𝜒^2 and df are used in the chi-square test for homogeneity of risk difference across studies. Z value is used in the normal Z test for whether the overall risk difference is zero. CI is the confidence interval.


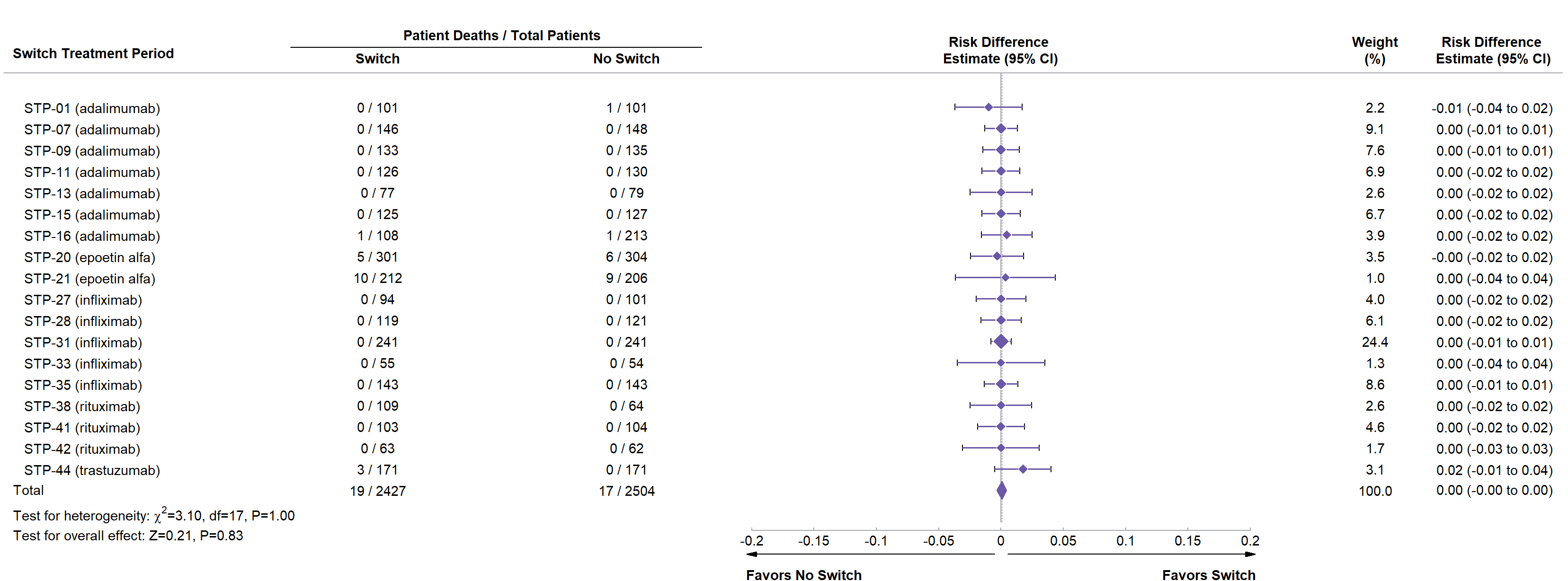


**Fig S19.** **Switching once from reference biologic to biosimilar - risk difference for severe adverse events.**

Meta-analysis was performed of the risk difference for one or more serious adverse events between Switch and No Switch arms in each switch treatment period (STP) containing a Switch arm where patients were switched once from a reference biologic to a biosimilar and a No Switch arm where patients remained on the reference biologic. Weight refers to the contribution of each STP to the overall estimate of risk difference, which is based on the inverse of the variance of the respective risk difference. 𝜒^2 and df are used in the chi-square test for homogeneity of risk difference across studies. Z value is used in the normal Z test for whether the overall risk difference is zero. CI is the confidence interval.


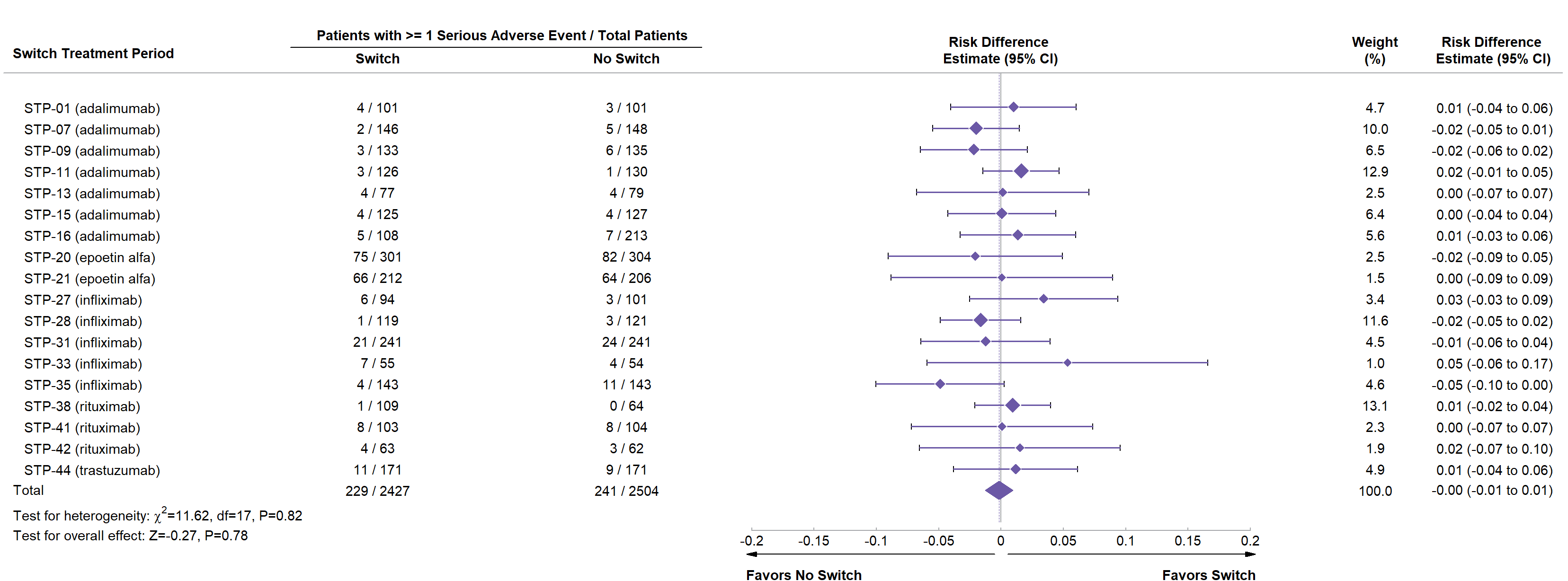


**Fig S20. Switching once from reference biologic to biosimilar - risk difference for discontinuation.**

Meta-analysis was performed of the risk difference for death between Switch and No Switch arms in each switch treatment period (STP) containing a Switch arm where patients were switched once from a reference biologic to a biosimilar and a No Switch arm where patients remained on the reference biologic. Weight refers to the contribution of each STP to the overall estimate of risk difference, which is based on the inverse of the variance of the respective risk difference. 𝜒^2 and df are used in the chi-square test for homogeneity of risk difference across studies. Z value is used in the normal Z test for whether the overall risk difference is zero. CI is the confidence interval.


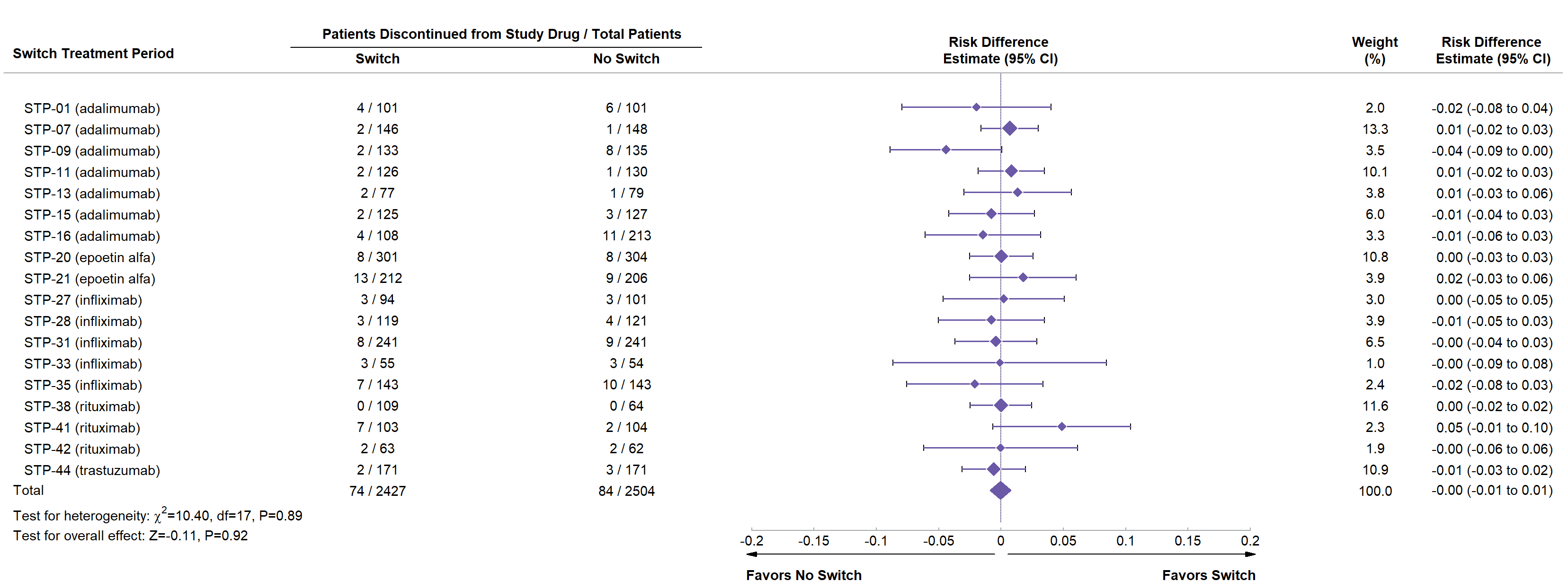


**Fig S21. Switching once from reference biologic to biosimilar or biosimilar to reference biologic - risk difference for death.**

Meta-analysis was performed of the risk difference for death between Switch and No Switch arms in each switch treatment period (STP) containing a Switch arm where patients were switched once from a reference biologic to a biosimilar or a biosimilar to a reference biologic and a No Switch arm where patients remained on the reference biologic or the biosimilar. Weight refers to the contribution of each STP to the overall estimate of risk difference, which is based on the inverse of the variance of the respective risk difference. 𝜒^2 and df are used in the chi-square test for homogeneity of risk difference across studies. Z value is used in the normal Z test for whether the overall risk difference is zero. CI is the confidence interval.


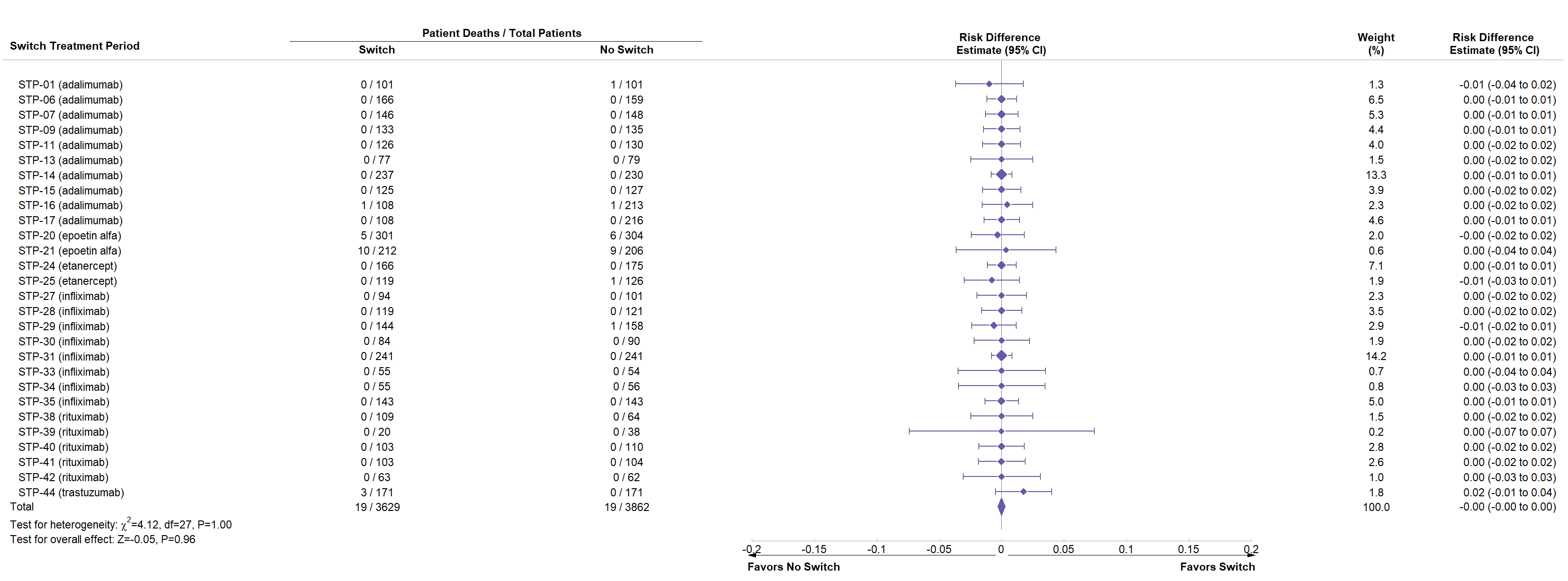


**Fig S22. Switching once from reference biologic to biosimilar or biosimilar to reference biologic - risk difference for severe adverse events.**

Meta-analysis was performed of the risk difference for one or more serious adverse events between Switch and No Switch arms in each switch treatment period (STP) containing a Switch arm where patients were switched once from a reference biologic to a biosimilar or a biosimilar to a reference biologic and a No Switch arm where patients remained on the reference biologic or the biosimilar. Weight refers to the contribution of each STP to the overall estimate of risk difference, which is based on the inverse of the variance of the respective risk difference. 𝜒^2 and df are used in the chi-square test for homogeneity of risk difference across studies. Z value is used in the normal Z test for whether the overall risk difference is zero. CI is the confidence interval.


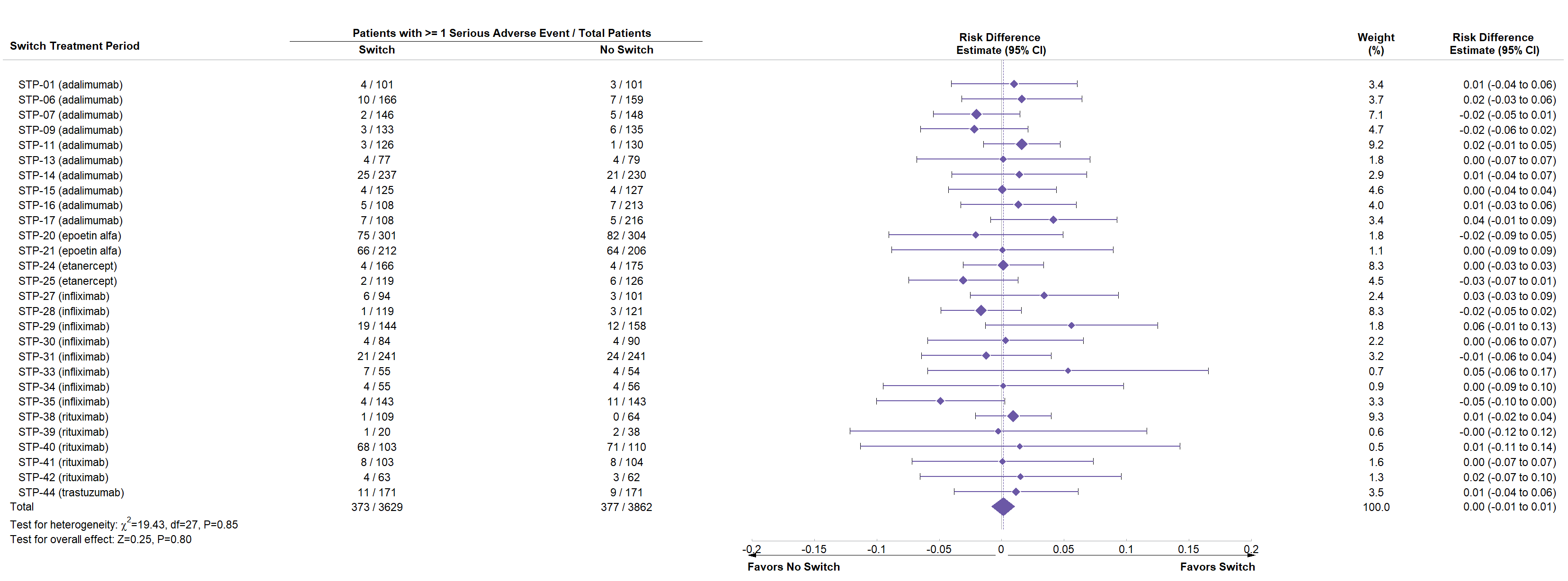


**Fig S23. Switching once from reference biologic to biosimilar or biosimilar to reference biologic - risk difference for discontinuation.**

Meta-analysis was performed of the risk difference for permanent discontinuation of study drug due to an adverse event between Switch and No Switch in each switch treatment period (STP) containing a Switch arm where patients were switched once from a reference biologic to a biosimilar or a biosimilar to a reference biologic and a No Switch arm where patients remained on the reference biologic or the biosimilar. Weight refers to the contribution of each STP to the overall estimate of risk difference, which is based on the inverse of the variance of the respective risk difference. 𝜒^2 and df are used in the chi-square test for homogeneity of risk difference across studies. Z value is used in the normal Z test for whether the overall risk difference is zero. CI is the confidence interval.


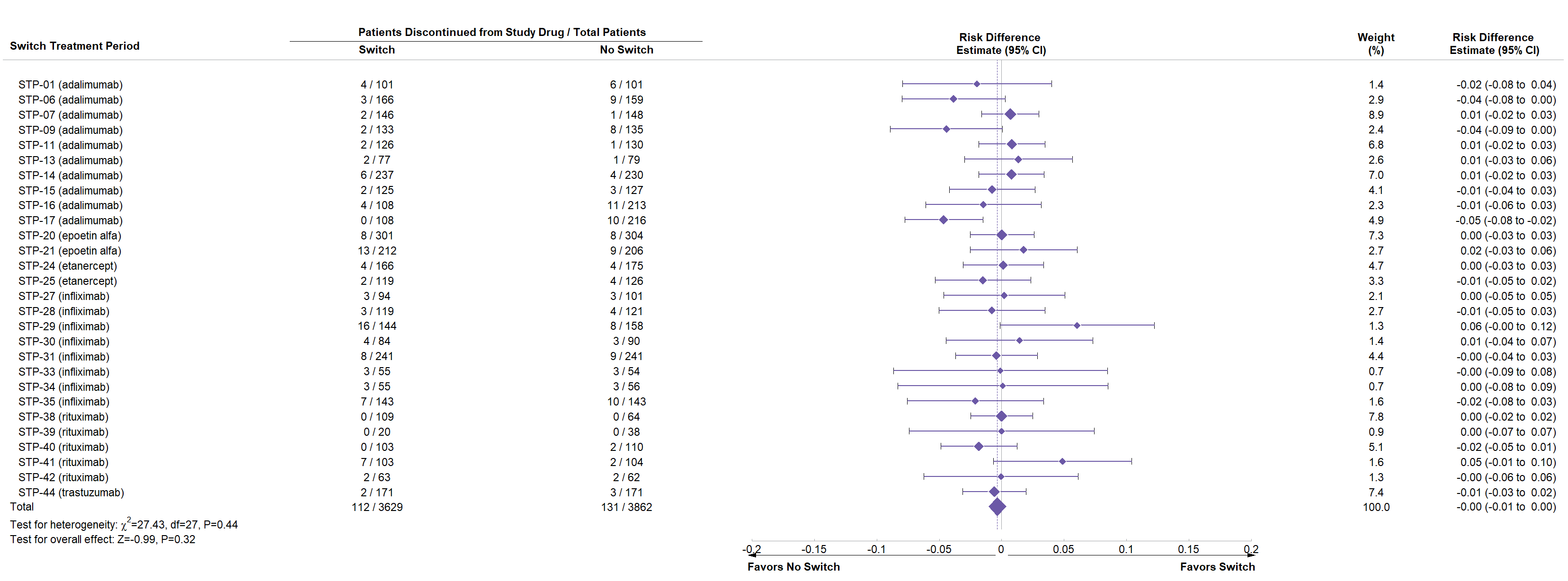


**Fig S24. Switching between reference biologic to biosimilar (multi-switch) - risk difference for death.**

Meta-analysis was performed of the risk difference for death between Switch and No Switch arms in each switch treatment period (STP) containing a Switch arm where patients were switched multiple times between a reference biologic and a biosimilar and a No Switch arm where patients remained on the reference biologic or the biosimilar. Weight refers to the contribution of each STP to the overall estimate of risk difference, which is based on the inverse of the variance of the respective risk difference. 𝜒^2 and df are used in the chi-square test for homogeneity of risk difference across studies. Z value is used in the normal Z test for whether the overall risk difference is zero. CI is the confidence interval.


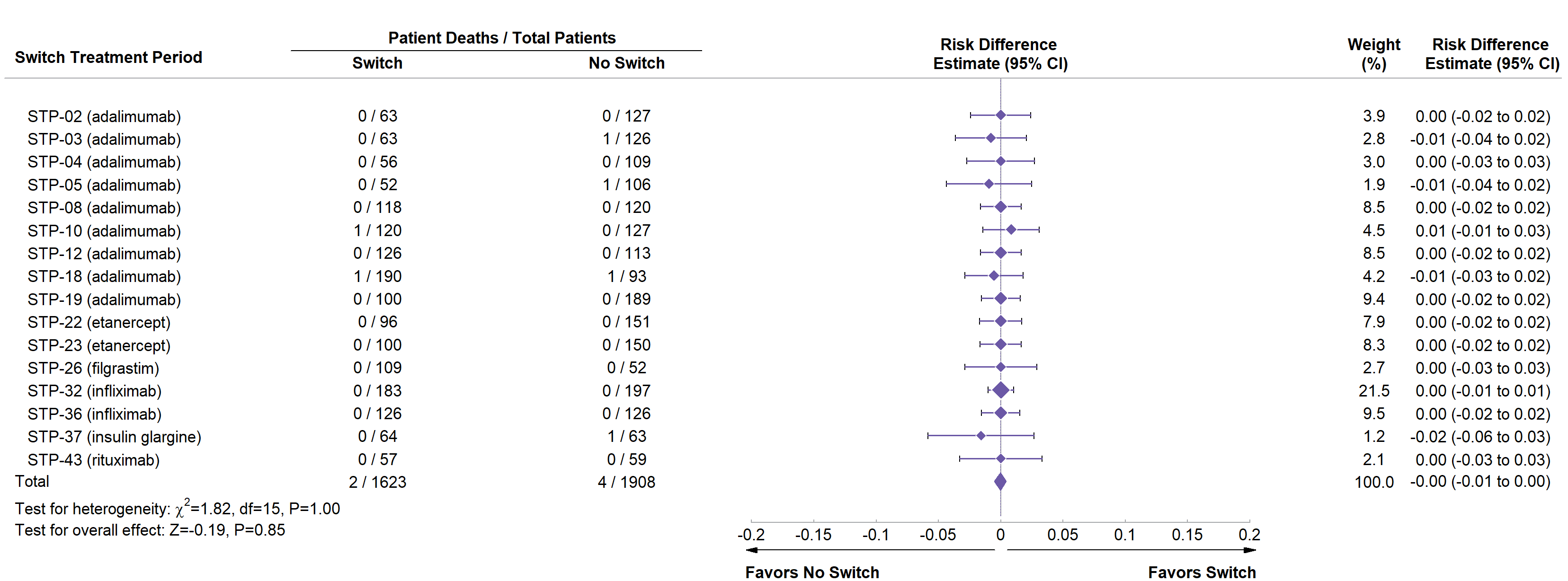


**Fig S25.** **Switching between reference biologic to biosimilar (multi-switch) - risk difference for severe adverse events.**

Meta-analysis was performed of the risk difference for one or more serious adverse events between Switch and No Switch arms in each switch treatment period (STP) containing a Switch arm where patients were switched multiple times between a reference biologic and a biosimilar and a No Switch arm where patients remained on the reference biologic or the biosimilar. Weight refers to the contribution of each STP to the overall estimate of risk difference, which is based on the inverse of the variance of the respective risk difference. 𝜒^2 and df are used in the chi-square test for homogeneity of risk difference across studies. Z value is used in the normal Z test for whether the overall risk difference is zero. CI is the confidence interval.


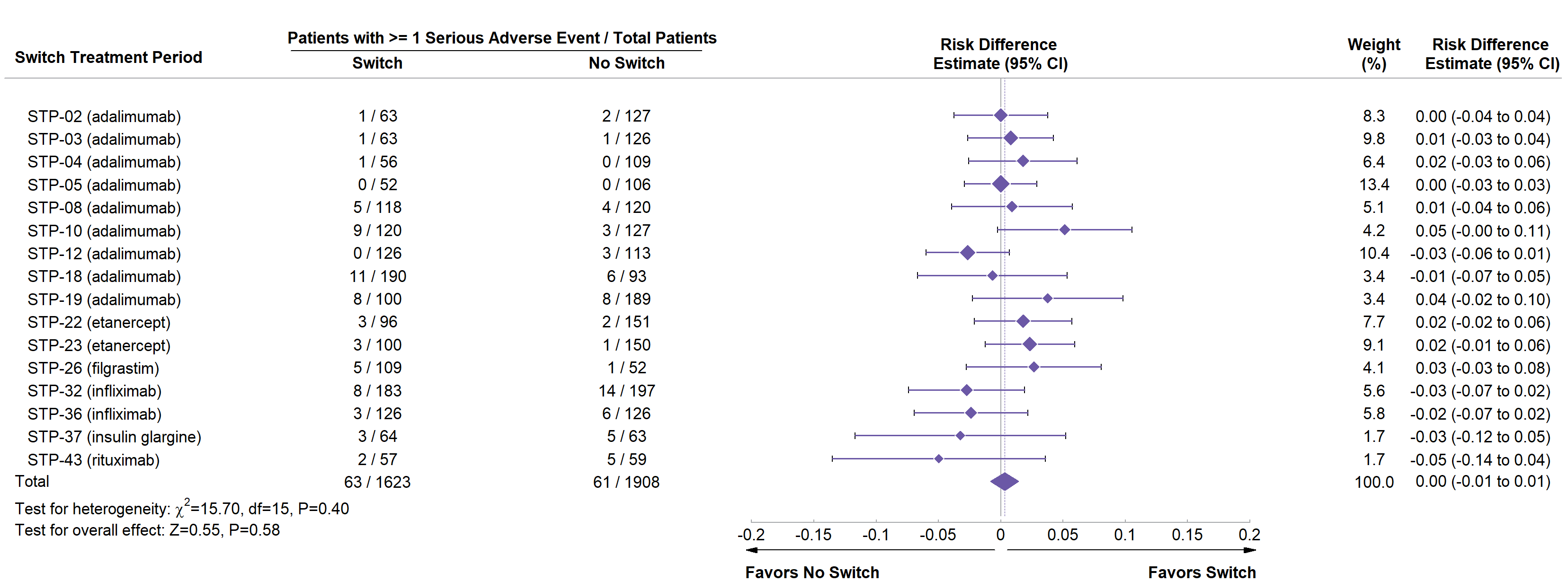


**Fig S26. Switching between reference biologic to biosimilar (multi-switch) - risk difference for discontinuation.**

Meta-analysis was performed of the risk difference for permanent discontinuation of study drug due to an adverse event between Switch and No Switch in each switch treatment period (STP) containing a Switch arm where patients were switched multiple times between a reference biologic and a biosimilar and a No Switch arm where patients remained on the reference biologic or the biosimilar. Weight refers to the contribution of each STP to the overall estimate of risk difference, which is based on the inverse of the variance of the respective risk difference. 𝜒^2 and df are used in the chi-square test for homogeneity of risk difference across studies. Z value is used in the normal Z test for whether the overall risk difference is zero. CI is the confidence interval.


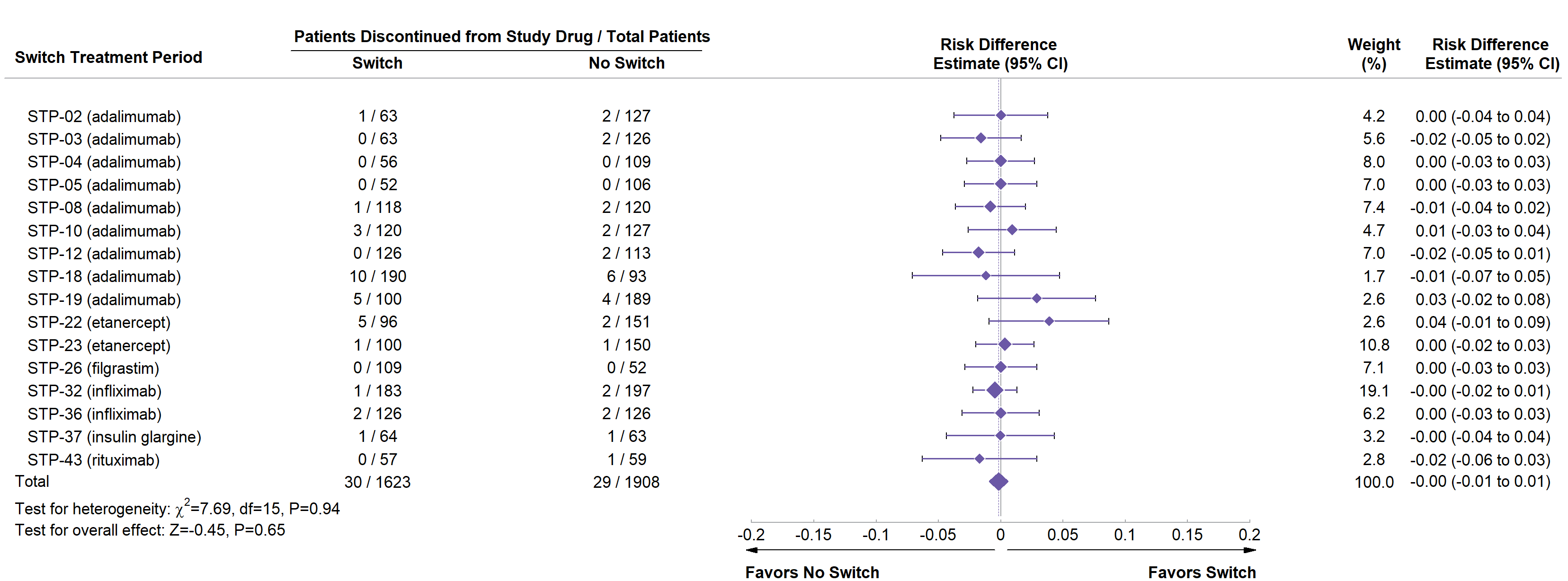


**Fig S27. Patients with antidrug antibody and neutralizing antibody positive assay results.**

Data collected at the end of a switch treatment period.


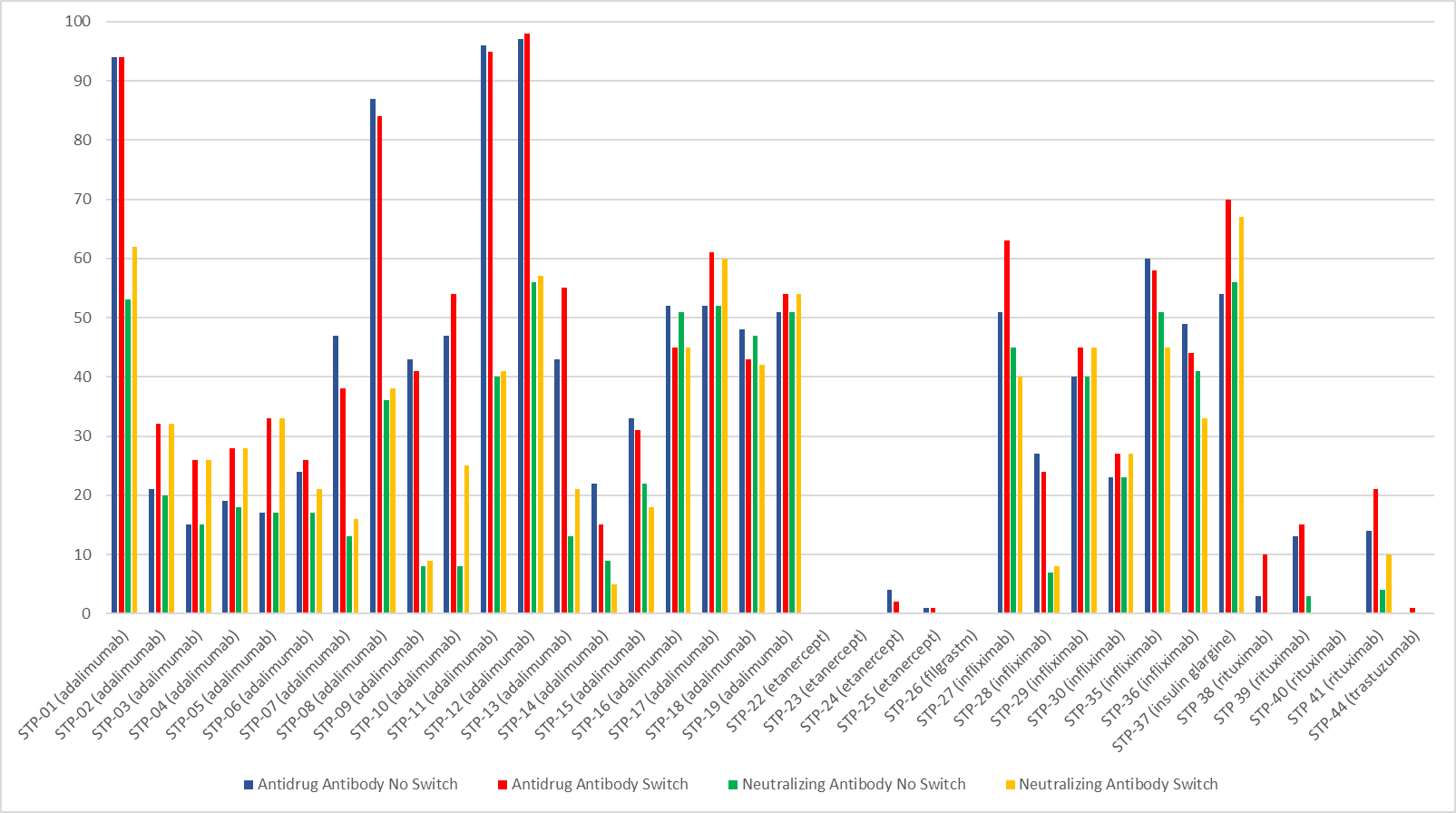


**Fig S28. Risk of bias.**

All STPs were reviewed for bias using a modified version of the Newcastle-Ottawa Assessment Scale (4, 6). STPs that scored 4 or more points on the modified Newcastle-Ottawa Assessment Scale were considered to have a low risk of bias (5, 6). Of the 44 STPs, 28 were found to be at low risk for bias, 8 STPs were found to be at lower risk of bias, and 8 STP were found to be at lowest risk for bias. The inability of reviewers to determine if the assessment of outcome was independent and blinded (Outcome 1) and determining the adequacy of follow-up (Outcome 3) were the most common reasons for a STP receiving a score less than 6. These were associated with STPs that were open label extensions of a parent randomized controlled trial.


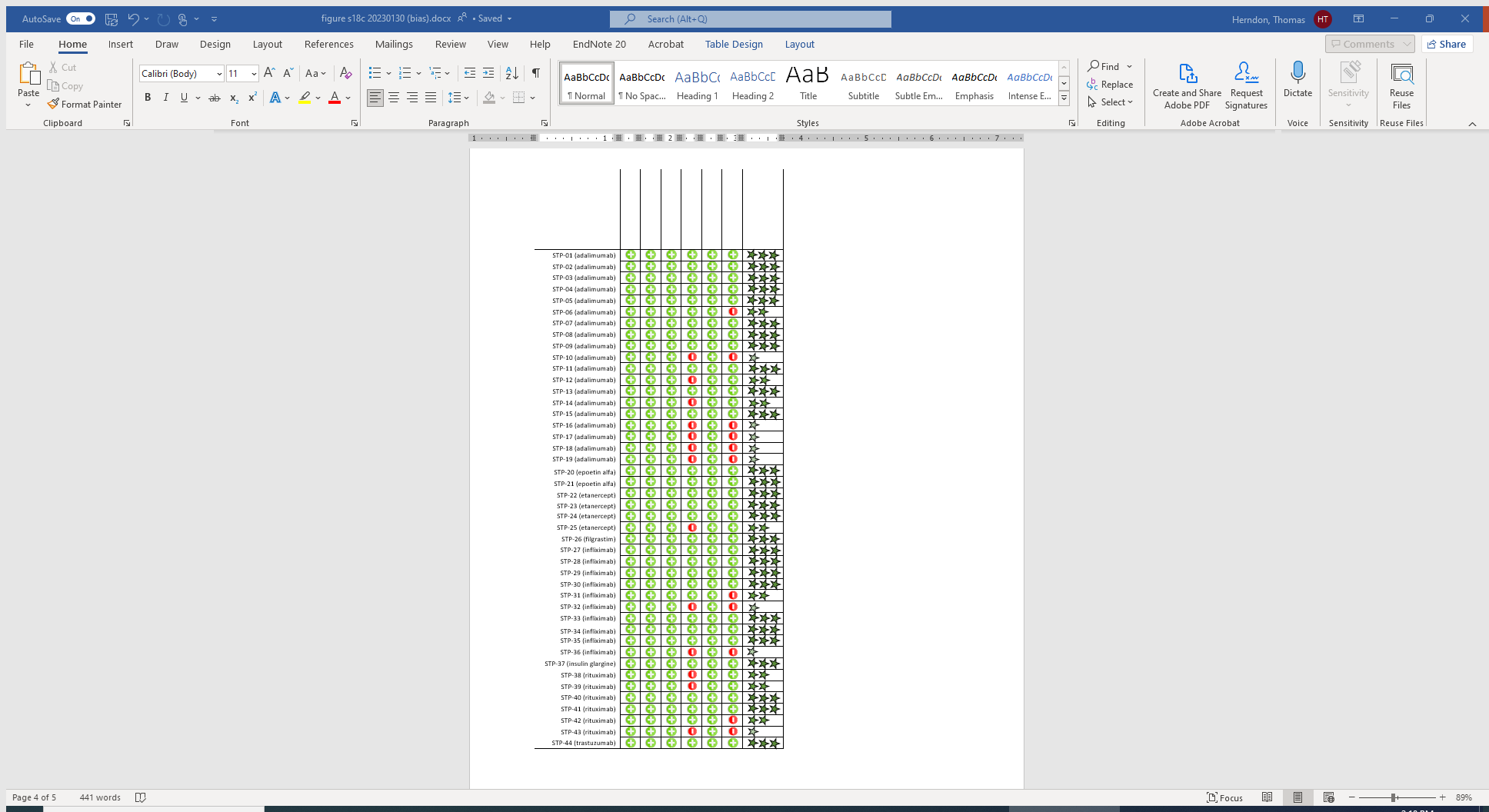


**Representativeness**

**of Switch Arm**

**Selection of**

**No Switch Arm**

**Outcome of Interest**

**Absent at STP Start**

**Assessment of**

**Outcome**

**Follow-up**

**(Length)**

**Follow-up**

**(Adequacy)**

**OVERALL**

**Switch Treatment Period (STP)**


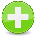

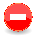


All elements present

Low risk of bias

Lower risk of bias

Lowest risk of bias

Less than all elements

present

**Supplementary tables**

**Table S1. Preferred Reporting Items for Systematic reviews and Meta-Analyses (PRISMA) checklist.**

Page numbers refer to the page number in the original submitted materials.

| **Section and Topic** | **Item #** | **Checklist item** | **Location where item is reported** |
| --- | --- | --- | --- |
| **TITLE** | | |  |
| Title | 1 | Identify the report as a systematic review. | 1 |
| **ABSTRACT** | | |  |
| Abstract | 2 | See the PRISMA 2020 for Abstracts checklist. | 2 |
| **INTRODUCTION** | | |  |
| Rationale | 3 | Describe the rationale for the review in the context of existing knowledge. | 3-4 |
| Objectives | 4 | Provide an explicit statement of the objective(s) or question(s) the review addresses. | 4-5 |
| **METHODS** | | |  |
| Eligibility criteria | 5 | Specify the inclusion and exclusion criteria for the review and how studies were grouped for the syntheses. | 5-6 |
| Information sources | 6 | Specify all databases, registers, websites, organisations, reference lists and other sources searched or consulted to identify studies. Specify the date when each source was last searched or consulted. | 5-6 |
| Search strategy | 7 | Present the full search strategies for all databases, registers and websites, including any filters and limits used. | 5,  Supporting information |
| Selection process | 8 | Specify the methods used to decide whether a study met the inclusion criteria of the review, including how many reviewers screened each record and each report retrieved, whether they worked independently, and if applicable, details of automation tools used in the process. | 5-6,  Supporting information |
| Data collection process | 9 | Specify the methods used to collect data from reports, including how many reviewers collected data from each report, whether they worked independently, any processes for obtaining or confirming data from study investigators, and if applicable, details of automation tools used in the process. | 6 |
| Data items | 10a | List and define all outcomes for which data were sought. Specify whether all results that were compatible with each outcome domain in each study were sought (e.g. for all measures, time points, analyses), and if not, the methods used to decide which results to collect. | 6 |
|  | 10b | List and define all other variables for which data were sought (e.g. participant and intervention characteristics, funding sources). Describe any assumptions made about any missing or unclear information. | 6  Supporting information n |
| Study risk of bias assessment | 11 | Specify the methods used to assess risk of bias in the included studies, including details of the tool(s) used, how many reviewers assessed each study and whether they worked independently, and if applicable, details of automation tools used in the process. | 6,  Supporting information |
| Effect measures | 12 | Specify for each outcome the effect measure(s) (e.g. risk ratio, mean difference) used in the synthesis or presentation of results. | 6-7 |
| Synthesis methods | 13a | Describe the processes used to decide which studies were eligible for each synthesis (e.g. tabulating the study intervention characteristics and comparing against the planned groups for each synthesis (item #5)). | 6-7 |
|  | 13b | Describe any methods required to prepare the data for presentation or synthesis, such as handling of missing summary statistics, or data conversions. | 6-7 |
|  | 13c | Describe any methods used to tabulate or visually display results of individual studies and syntheses. | Supporting information |
|  | 13d | Describe any methods used to synthesize results and provide a rationale for the choice(s). If meta-analysis was performed, describe the model(s), method(s) to identify the presence and extent of statistical heterogeneity, and software package(s) used. | 6-7 |
|  | 13e | Describe any methods used to explore possible causes of heterogeneity among study results (e.g. subgroup analysis, meta-regression). | Supporting information |
|  | 13f | Describe any sensitivity analyses conducted to assess robustness of the synthesized results. | NA |
| Reporting bias assessment | 14 | Describe any methods used to assess risk of bias due to missing results in a synthesis (arising from reporting biases). | NA |
| Certainty assessment | 15 | Describe any methods used to assess certainty (or confidence) in the body of evidence for an outcome. | 19-20,  Supporting information |
| **RESULTS** | | |  |
| Study selection | 16a | Describe the results of the search and selection process, from the number of records identified in the search to the number of studies included in the review, ideally using a flow diagram. | 7-9,  Fig 1 |
|  | 16b | Cite studies that might appear to meet the inclusion criteria, but which were excluded, and explain why they were excluded. | Fig 1 |
| Study characteristics | 17 | Cite each included study and present its characteristics. | 7, 13,  Table 1,  Supporting information |
| Risk of bias in studies | 18 | Present assessments of risk of bias for each included study. | 16,  Supporting information n |
| Results of individual studies | 19 | For all outcomes, present, for each study: (a) summary statistics for each group (where appropriate) and (b) an effect estimate and its precision (e.g. confidence/credible interval), ideally using structured tables or plots. | 15-16, 19-20,  Supporting information |
| Results of syntheses | 20a | For each synthesis, briefly summarise the characteristics and risk of bias among contributing studies. | 17,  Supporting information |
|  | 20b | Present results of all statistical syntheses conducted. If meta-analysis was done, present for each the summary estimate and its precision (e.g. confidence/credible interval) and measures of statistical heterogeneity. If comparing groups, describe the direction of the effect. | 15-16, 19-20,  Supporting information |
|  | 20c | Present results of all investigations of possible causes of heterogeneity among study results. | 19-20,  Supporting information |
|  | 20d | Present results of all sensitivity analyses conducted to assess the robustness of the synthesized results. | NA |
| Reporting biases | 21 | Present assessments of risk of bias due to missing results (arising from reporting biases) for each synthesis assessed. | 12,  Supporting information |
| Certainty of evidence | 22 | Present assessments of certainty (or confidence) in the body of evidence for each outcome assessed. | 15-16,  Supporting information |
| **DISCUSSION** | | |  |
| Discussion | 23a | Provide a general interpretation of the results in the context of other evidence. | 17 |
|  | 23b | Discuss any limitations of the evidence included in the review. | 19-20 |
|  | 23c | Discuss any limitations of the review processes used. | 19-20 |
|  | 23d | Discuss implications of the results for practice, policy, and future research. | 20 |
| **OTHER INFORMATION** | | |  |
| Registration and protocol | 24a | Provide registration information for the review, including register name and registration number, or state that the review was not registered. | NA |
|  | 24b | Indicate where the review protocol can be accessed, or state that a protocol was not prepared. | NA |
|  | 24c | Describe and explain any amendments to information provided at registration or in the protocol. | NA |
| Support | 25 | Describe sources of financial or non-financial support for the review, and the role of the funders or sponsors in the review. | Financial Disclosure Statement at submission |
| Competing interests | 26 | Declare any competing interests of review authors. | Provided at submission |
| Availability of data, code and other materials | 27 | Report which of the following are publicly available and where they can be found: template data collection forms; data extracted from included studies; data used for all analyses; analytic code; any other materials used in the review. | Supporting information |

NA, Not applicable

**Table S2. Additional information on included switch treatment periods.**

| **Switch Treatment Period** | **Source**  **(Includes Biosimilar Product Nonproprietary Name)** | **National Clinical Trial Number** | **Design** | **Patient Population** | **Treatment Before STP (Weeks)** | **STP Length (Weeks)** | **Switches up to and Including STP**  **(n)** | **Randomization Ratio (No Switch: Switch)** | **Full Switch Pattern:**  **No Switch  (Control Arm, n)** | **Full Switch Pattern:**  **Switch  (Test Arm, n)** |
| --- | --- | --- | --- | --- | --- | --- | --- | --- | --- | --- |
| **adalimumab** |  |  |  |  |  |  |  |  |  |  |
| STP-01 | FDA Reviews of adalimumab-aacf (7) | NCT02660580 | RDB | PS | 16 | 52 | 1 | 1:1 | R-R, 101 | R-B, 101 |
| STP-02^a^ | FDA Reviews of adalimumab-adaz (8) | NCT02016105 | RDB | PS | 17 | 18 | 3 | 2:1 | R-R-R-R, 127 | R-B-R-B, 63 |
| STP-03^a^ | FDA Reviews of adalimumab-adaz (8) | NCT02016105 | RDB | PS | 17 | 18 | 3 | 2:1 | B-B-B-B, 126 | B-R-B-R, 63 |
| STP-04^a^ | FDA Reviews of adalimumab-adaz (8) | NCT02016105 | RDB | PS | 35 | 16 | 4 | 2:1 | R-R-R-R, 109 | B-R-B-R-B, 56 |
| STP-05^a^ | FDA Reviews of adalimumab-adaz (8) | NCT02016105 | RDB | PS | 35 | 16 | 4 | 2:1 | B-B-B-B-B, 106 | R-B-R-B-R, 52 |
| STP-06 | Wiland 2020 (9)  (adalimumab-adaz) | NCT02744755 | RDB | RA | 24 | 24 | 1 | 1:1 | B-B, 159 | R-B, 166 |
| STP-07 | FDA Reviews of adalimumab-adbm (10) | NCT02137226 | RDB | RA | 24 | 24 | 1 | 1:1 | R-R, 148 | R-B, 146 |
| STP-08 | FDA Reviews of adalimumab-adbm (10) | NA | RDB | PS | 14 | 18 | 3 | 1:1 | R-R-R-R, 120 | R-B-R-B, 118 |
| STP-09 | FDA Reviews of adalimumab-afzb (11) | NCT02480153 | RDB | RA | 26 | 26 | 1 | 1:1 | R-R, 135 | R-B, 133 |
| STP-10 | FDA reviews of adalimumab-afzb (11) Fleischmann 2021 (12) | NCT02480153 | OLE | RA | 52 | 26 | 2 | 1:1 | R-B-B, 127 | R-R-B, 120 |
| STP-11 | FDA Reviews of adalimumab-aqvh (13) | NCT02489227 | RDB | PS | 16 | 8 | 1 | 1:1 | R-R, 130 | R-B, 126 |
| STP-12 | FDA Reviews of adalimumab-aqvh (13) | NCT02489227 | OLE | PS | 24 | 24 | 2 | 1:1 | R-B-B, 113 | R-R-B, 126 |
| STP-13 | FDA Reviews of adalimumab-atto (14) Papp 2017 (15) | NCT01970488 | RDB | PS | 16 | 32 | 1 | 1:1 | R-R, 79 | R-B, 77 |
| STP-14 | Cohen 2019 (16)  (adalimumab-atto) | NCT02114931 | OLE | RA | 22 | 72 | 1 | 1:1 | B-B, 230 | R-B, 237 |
| STP-15 | FDA Reviews of adalimumab-bwwd (17) Weinblatt 2018 (18) | NCT02167139 | RDB | RA | 24 | 26 | 1 | 1:1 | R-R, 127 | R-B, 125 |
| STP-16 | FDA Reviews of adalimumab-fkjp (19) | NCT02405780 | OLE | RA | 24 | 30 | 1 | 2:1 | R-R, 213 | R-B, 108 |
| STP-17 | FDA Reviews of adalimumab-fkjp (19) | NCT02405780 | OLE | RA | 24 | 30 | 1 | 2:1 | B-B, 216 | B-R, 108 |
| STP-18 | FDA Reviews of adalimumab-fkjp(19) | NCT02405780 | OLE | RA | 54 | 49 | 2 | 1:2 | R-B-B, 93 | R-R-B, 190 |
| STP-19 | FDA reviews of adalimumab-fkjp (19) | NCT02405780 | OLE | RA | 54 | 49 | 2 | 2:1 | B-B-B, 189 | B-R-B, 100 |
| **epoetin alfa** |  |  |  |  |  |  |  |  |  |  |
| STP-20 | FDA Reviews of epoetin alfa-epbx (20) Fishbane 2018(21) | NCT01473407 | RDB | CKD | 4 | 24 | 1 | 1:1 | R-R, 304 | R-B, 301 |
| STP-21 | Thadhani 2018 (22)  (epoetin alfa-epbx) | NCT02504294 | ROL | CKD | ≥ 16 | 24 | 1 | 1:1 | R-R, 206 | R-B, 212 |
| **etanercept** |  |  |  |  |  |  |  |  |  |  |
| STP-22 | FDA Reviews of etanercept-szzs (23) | NCT01891864 | RDB | PS | 12 | 18 | 3 | 3:2 | R-R-R-R, 151 | R-B-R-B, 96 |
| STP-23 | FDA Reviews of etanercept-szzs (23) | NCT01891864 | RDB | PS | 12 | 18 | 3 | 3:2 | B-B-B-B, 150 | B-R-B-R, 100 |
| STP-24 | Jaworski 2019 (24)  (etanercept-szzs) | NCT02638259 | RDB | RA | 24 | 24 | 1 | 1:1 | B-B, 175 | R-B, 166 |
| STP-25 | FDA Reviews of etanercept-ykro (25) Emery 2017 (26) | NCT01895309 | OLE | RA | 52 | 48 | 1 | 1:1 | B-B, 126 | R-B, 119 |
| **filgrastim** |  |  |  |  |  |  |  |  |  |  |
| STP-26 | Blackwell 2018 (27)  (filgrastim-sndz) | NCT01519700 | RDB | BC | 3 | 15 | 5 | 1:2 | R-R-R-R-R-R, 52 | R-B-R-B-R-B and   B-R-B-R-B-R, 109 |
| **Infliximab** |  |  |  |  |  |  |  |  |  |  |
| STP-27 | FDA Reviews of infliximab-adba (28) | NCT01936181 | RDB | RA | 54 | 24 | 1 | 1:1 | R-R, 101 | R-B, 94 |
| STP-28 | FDA Reviews of infliximab-axxq (29) | NCT02937701 | RDB | RA | 22 | 24 | 1 | 1:1 | R-R, 121 | R-B, 119 |
| STP-29 | FDA Reviews of infliximab-dyyb (30) Yoo 2017 (31) | NCT01571219 | Blinded to previous treatment | RA | 54 | 48 | 1 | 1:1 | B-B, 158 | R-B, 144 |
| STP-30 | FDA Reviews of infliximab-dyyb (30) Park 2017 (32) | NCT01571206 | OLE | AS | 54 | 48 | 1 | 1:1 | B-B, 90 | R-B, 84 |
| STP-31 | Jørgensen 2017 (33)  (infliximab-dyyb) | NCT02148640 | RDB | CD, UC, SA, RA, PA, PS | ≥ 24 | 52 | 1 | 1:1 | R-R, 241 | R-B, 241 |
| STP-32 | Goll 2019 (34)  (infliximab-dyyb) | NCT02148640 | RDB | CD, UC, SA, RA, PA, PS | 52 | 26 | 2 | 1:1 | R-B-B, 197 | R-R-B, 183 |
| STP-33 | Ye 2019 (35)  (infliximab-dyyb) | NCT02096861 | RDB | CD | 30 | 24 | 1 | 1:1 | R-R, 54 | R-B, 55 |
| STP-34 | Ye 2019 (35)  (infliximab-dyyb) | NCT02096861 | RDB | CD | 30 | 24 | 1 | 1:1 | B-B, 56 | B-R, 55 |
| STP-35 | FDA Reviews of infliximab-qbtx (36) | NCT02222493 | RDB | RA | 30 | 54 | 1 | 1:1 | R-R, 143 | R-B, 143 |
| STP-36 | Cohen 2020 (37)  (infliximab-qbtx) | NCT02222493 | OLE | RA | 54 | 24 | 2 | 1:1 | R-B-B, 126 | R-R-B, 126 |
| **insulin glargine** |  |  |  |  |  |  |  |  |  |  |
| STP-37 | FDA Reviews of insulin glargine-yfgn (38) | NCT02666430 | ROL | DM | 52 | 36 | 3 | 1:1 | R-R-R-R, 63 | R-B-R-B, 64 |
| **Rituximab** |  |  |  |  |  |  |  |  |  |  |
| STP-38^a^ | FDA Reviews of rituximab-abbs(39) Shim 2019 (40) | NCT02149121 | ROL | RA | 48 | 24 | 1 | 1:1 | R-R, 64 | R-B, 109 |
| STP-39 | FDA Reviews of rituximab-abbs (39) | NCT01873443 | ROL | RA | 24 to 48 | 24 | 1 | 1:1 | B-B, 38 | R-B, 20 |
| STP-40 | Kwak 2022 (41, 42)  (rituximab-abbs) | NCT02260804 | RDB | FL | 64 | 52 | 1 | 1:1 | B-B, 110 | R-B, 103 |
| STP-41^a^ | FDA Reviews of rituximab-arrx (43) | NCT02792699 | ROL | RA | 24 | 24 | 1 | 1:1 | R-R, 104 | R-B, 103 |
| STP-42^a^ | FDA Reviews of rituximab-pvvr (44) | NCT01643928 | ROL | RA | ≥ 16 | 9 | 1 | 1:1 | R-R, 62 | R-B, 63 |
| STP-43^a^ | FDA Reviews of rituximab-pvvr (44) Cohen 2018 (45) | NCT01643928 | Blinded random-ization | RA | ≥ 16 | ≥ 16 | 2 | 1:1 | R-B-B, 59 | R-R-B, 57 |
| **trastuzumab** |  |  |  |  |  |  |  |  |  |  |
| STP-44 | FDA Reviews of trastuzumab-anns (46) von Minckwitz 2018(47) | NCT01901146 | RDB | BC | ≤ 12 | ≤ 33 | 1 | 1:1 | R-R, 171 | R-B, 171 |

AS, Ankylosing Spondylitis; B, Biosimilar Product; BC, Breast Cancer; CD, Crohn Disease; CKD, Chronic Kidney Disease; DM, Type 1 Diabetes Mellitus; FL, Follicular Lymphoma; NCT, National Clinical Trial Number; NA, Not Applicable; OLE, Open Label Extension; PA, Psoriatic Arthritis; PS, Plaque Psoriasis; RA, Rheumatoid Arthritis; RDB, Randomized Double Blind; ROL, Randomized Open Label; SA, Spondylarthritis; STP, Switch Treatment Period; UC, Ulcerative Colitis.

^a^Sufficient data included in the submission to justify combining EU and US Reference Biologic arms.

**Table S3. Demographics (age, sex, body mass index) of patients in switch treatment periods.**

Data collected from time of entry into STP unless noted.

| **Characteristic** |  | **STP-01^a^ adalimumab** | **STP-02^b,c^ adalimumab** | **STP-03^b,c^ adalimumab** | **STP-04^b,c^ adalimumab** | **STP-05^b,c^ adalimumab** |
| --- | --- | --- | --- | --- | --- | --- |
| **Age (years) (Mean (SD); Min, Max)** | **No Switch** | 43 (12.23); 43; 21, 74 | 47 (0.78); 18, 81 | 47 (0.78); 18, 81 | 47 (0.78); 18, 81 | 47 (0.78); 18, 81 |
|  | **Switch** | 42 (11.90); 41; 22, 69 | 47 (1.61); 19, 80 | 47 (1.61); 19, 80 | 47 (1.61); 19, 80 | 47 (1.61); 19, 80 |
| **Sex (Percent Female, %)** | **No Switch** | 38 | 36 | 36 | 36 | 36 |
|  | **Switch** | 28 | 44 | 44 | 44 | 44 |
| **BMI (kg/m2) (Mean (SD); Min, Max)** | **No Switch** | 26 (3.13); 20, 39 | 31 (0.23); 17, 61 | 31 (0.23); 17, 61 | 31 (0.23); 17, 61 | 31 (0.23); 17, 61 |
|  | **Switch** | 26 (3.18); 18, 31 | 31 (0.46); 17, 56 | 31 (0.46); 17, 56 | 31 (0.46); 17, 56 | 31 (0.46); 17, 56 |

| **Characteristic** |  | **STP-06 adalimumab** | **STP-07 adalimumab** | **STP-08 adalimumab** | **STP-09 adalimumab** | **STP-10 adalimumab** |
| --- | --- | --- | --- | --- | --- | --- |
| **Age (years) (Mean (SD); Min, Max)** | **No Switch** | 53 (13); NR, NR | 53 (12); NR, NR | 44 (NR); 19, 76 | 54 (12); 24, 78 | 53 (14); NR, NR |
|  | **Switch** | 54 (12); NR, NR | 54 (10); NR, NR | 46 (NR); 19, 76 | 53 (14); 18, 79 | 53 (12); NR, NR |
| **Sex (Percent Female, %)** | **No Switch** | 85 | 87 | 37 | 80 | 69 |
|  | **Switch** | 80 | 82 | 31 | 71 | 81 |
| **BMI (kg/m2) (Mean (SD); Min, Max)** | **No Switch** | 29 (7); NR, NR | 27 (6); NR, NR | 31 (7); NR, NR | 28 (7); 16, 56 | 27 (6); NR, NR |
|  | **Switch** | 28 (6); NR, NR | 28 (6); NR, NR | 30 (6); NR, NR | 28 (6); 18, 49 | 29 (8); NR, NR |

| **Characteristic** |  | **STP-11 adalimumab** | **STP-12 adalimumab** | **STP-13 adalimumab** | **STP-14 adalimumab** | **STP-15 adalimumab** |
| --- | --- | --- | --- | --- | --- | --- |
| **Age (years) (Mean (SD); Min, Max)** | **No Switch** | 44 (12); NR, NR | 45 (14); NR, NR | NR (NR); NR, NR (median=39) | 55 (12); NR, NR | 53 (12); NR, NR |
|  | **Switch** | 45 (14); NR, NR | 44 (12); NR, NR | NR (NR); NR, NR (median=46) | 56 (11); NR, NR | 52 (11); NR, NR |
| **Sex (Percent Female, %)** | **No Switch** | 28 | 23 | 33 | 82 | 80 |
|  | **Switch** | 23 | 28 | 31 | 81 | 84 |
| **BMI (kg/m2) (Mean (SD); Min, Max)** | **No Switch** | 29 (6): NR, NR | 29 (6); NR, NR | 30 (6); 20, 50 | NR (NR); NR, NR | 27 (5); NR, NR |
|  | **Switch** | 30 (6); NR, NR | 29 (6): NR, NR | 29 (5); 20, 48 | NR (NR); NR, NR | 27 (5); NR, NR |

| **Characteristic** |  | **STP-16 adalimumab** | **STP-17 adalimumab** | **STP-18_d_ adalimumab** | **STP-19^e^ adalimumab** | **STP-20 epoetin-alfa** |
| --- | --- | --- | --- | --- | --- | --- |
| **Age (years) (Mean (SD); Min, Max)** | **No Switch** | 54 (13); 21, 93 | 53 (12); 18, 85 | 52 (12); 23, 82 | 53 (12); 18, 85 | NR (NR); 25, 80  (median=58) |
|  | **Switch** | 52 (12); 23, 82 | 52 (11); 24, 77 | 54 (13); 21, 93 | 52 (11): 24, 77 | NR (NR); 21, 78  (median=57) |
| **Sex (Percent Female, %)** | **No Switch** | 80 | 75 | 77 | 75 | 42 |
|  | **Switch** | 77 | 79 | 80 | 79 | 48 |
| **BMI (kg/m2) (Mean (SD); Min, Max)** | **No Switch** | NR (NR); NR, NR  (weight (kg)=74 (15); 41, 122) | NR (NR); NR, NR  (weight (kg)=74 (16); 41, 116) | NR (NR); NR, NR  (weight (kg)=75 (17); 44, 115) | NR (NR); NR, NR  (weight (kg)=74 (16); 41, 116 | 31 (8); 17, 96 |
|  | **Switch** | NR (NR); NR, NR  (weight (kg)=75 (17); 44, 115 | NR (NR); NR, NR  (weight (kg)=75 (16); 43, 123) | NR (NR); NR, NR  (weight (kg)=74 (15); 41, 122) | NR (NR); NR, NR  (weight (kg)=75 (16); 43, 123) | 31 (9); 15, 90 |

| **Characteristic** |  | **STP-21 epoetin-alfa** | **STP-22^f,g^ etanercept** | **STP-23^f,g^ etanercept** | **STP-24 etanercept** | **STP-25 etanercept** |
| --- | --- | --- | --- | --- | --- | --- |
| **Age (years) (Mean (SD); Min, Max)** | **No Switch** | 59 (14); 21, 90 | 43 (13); 19, 71 | 42 (12); 18, 75 | 55 (11); NR, NR | 50 (12); NR, NR |
|  | **Switch** | 61 (14); 26, 94 | 42 (12); 18, 78 | 42 (12); 18, 78 | 52 (13); NR, NR | 52 (11); NR, NR |
| **Sex (Percent Female, %)** | **No Switch** | 50 | 36 | 37 | 85 | 85 |
|  | **Switch** | 39 | 40 | 40 | 79 | 84 |
| **BMI (kg/m2) (Mean (SD); Min, Max)** | **No Switch** | 30 (8);17, 73 | 29 (5); 18, 47 | 28(6); 17, 48 | NR (NR); NR, NR | 27 (6); NR, NR |
|  | **Switch** | 31 (8); 16, 66 | 29 (6); 17, 47 | 29 (6); 17, 47 | NR (NR); NR, NR | 26 (5); NR, NR |

| **Characteristic** |  | **STP-26 filgrastim** | **STP-27 infliximab** | **STP-28 infliximab** | **STP-29 infliximab** | **STP-30 infliximab** |
| --- | --- | --- | --- | --- | --- | --- |
| **Age (years) (Mean (SD); Min, Max)** | **No Switch** | 47 (11); 23, 76 | 51 (11); NR, NR | 55 (11); 27, 75 | 49 (10); NR, NR | 38 (12); 18, 69 |
|  | **Switch** | 49 (11); 26, 73 | 53 (11); NR, NR | 54 (12); 19, 77 | 49 (11); NR, NR | 39 (12); 18, 66 |
| **Sex (Percent Female, %)** | **No Switch** | 100 | 78 | 74 | 79 | 22 |
|  | **Switch** | 100 | 82 | 83 | 84 | 14 |
| **BMI (kg/m2) (Mean (SD); Min, Max)** | **No Switch** | NR (NR); NR, NR | 27 (6); NR, NR | 28 (6); 17, 52 | 27 (NR); 17, 50 | 24 (NR); 18, 39 |
|  | **Switch** | NR (NR); NR, NR | 26 (5); NR, NR | 28 (6); 18, 47 | 26 (NR); 17, 45 | 26 (NR); 18, 42 |

| **Characteristic** |  | **STP-31 infliximab** | **STP-32 infliximab** | **STP-33^g^ infliximab** | **STP-34^g^ infliximab** | **STP-35 infliximab** |
| --- | --- | --- | --- | --- | --- | --- |
| **Age (years) (Mean (SD); Min, Max)** | **No Switch** | 48 (15); NR, NR | 49 (15); NR, NR | R: NR (NR); 24, 45  (median=32)  B: NR (NR); 26, 46  (median=35) | R: NR (NR); 24, 45  (median=32)  B: NR (NR); 26, 46  (median=35) | 53 (13); NR, NR |
|  | **Switch** | 48 (15); NR, NR | 48 (14); NR, NR | NR (NR); NR, NR | NR (NR); NR, NR | 53 (13); NR, NR |
| **Sex (Percent Female, %)** | **No Switch** | 41 | 33 | R: 45  B: 43 | R: 45  B: 43 | 78 |
|  | **Switch** | 36 | 43 | NR | NR | 83 |
| **BMI (kg/m2) (Mean (SD); Min, Max)** | **No Switch** | NR (NR); NR, NR | NR (NR); NR, NR | NR (NR); NR, NR | NR (NR); NR, NR | 27 (7); NR, NR |
|  | **Switch** | NR (NR); NR, NR | NR (NR); NR, NR | NR (NR); NR, NR | NR (NR); NR, NR | 28 (7); NR, NR |

| **Characteristic** |  | **STP-36 infliximab** | **STP-37 insulin-glargine** | **STP-38^b^ rituximab** | **STP-39 rituximab** | **STP-40 rituximab** |
| --- | --- | --- | --- | --- | --- | --- |
| **Age (years) (Mean (SD); Min, Max)** | **No Switch** | 51 (13); NR; NR, NR | 43 (13); 44; 20, 66 | 52(10); NR, NR | 51 (11); NR, NR | 58 (13); NR, NR |
|  | **Switch** | 54 (12); NR; NA, NA | 45 (11); 45; 20, 66 | 52(11); NR, NR | 50 (11); NR, NR | 58 (12); NR, NR |
| **Sex (Percent Female, %)** | **No Switch** | 83 | 43 | 84 | 92 | 49 |
|  | **Switch** | 78 | 36 | 89 | 90 | 55 |
| **BMI (kg/m2) (Mean (SD); Min, Max)** | **No Switch** | 28 (7); NR, NR | 27 (4); 19, 36 | 27 (4); NR, NR | 28 (7); NR, NR | NR (NR); NR, NR |
|  | **Switch** | 27 (7); NR, NR | 27 (4); 20, 37 | 28 (6); NR, NR | 28 (5); NR, NR | NR (NR); NR, NR |

| **Characteristic** |  | **STP-41^b^ rituximab** | **STP-42^b,c^ rituximab** | **STP-43^b^ rituximab** | **STP-44 trastuzumab** |
| --- | --- | --- | --- | --- | --- |
| **Age (years) (Mean (SD); Min, Max)** | **No Switch** | 57 (11); 24, 79 | 54 (0.90); 20, 80 | 55 (0.13); 26, 81 | 52 (11); 26, 79 |
|  | **Switch** | 56 (11); 27, 77 | NR (NR); NR, NR | 56 (0.73); 34, 82 | 53 (12); 28, 79 |
| **Sex (Percent Female, %)** | **No Switch** | 88 | 76 | 79 | 100 |
|  | **Switch** | 81 | NR | 76 | 100 |
| **BMI (kg/m2) (Mean (SD); Min, Max)** | **No Switch** | 29 (7); 17, 55 | 29 (0.29); 17, 47 | 30 (0.72); 16, 43 | NR (NR); NR, NR  (weight (kg) 70 (NR); 62, 79) |
|  | **Switch** | 28 (6); 16, 51 | NR (NR); NR, NR | 29 (0.48); 17, 41 | NR (NR); NR, NR  (weight (kg) 73 (NR); 62, 81) |

BMI, Body Mass Index; B, Biosimilar Product; NR, Not Reported; R, Reference Biologic; STP, Switch Treatment Period

^a^Baseline data for No Switch arm is from R at time of entry into study.

^b^Sufficient data included in the submission to justify combining E.U. and U.S. Reference Biologic arms.

^c^Baseline data at time of entry into study

^d^Baseline data at time of entry into STP-16

^e^Baseline data at time of entry into STP-17

^f^Baseline data for switch arm is pooled for both switch arms (R-B and B-R).

^g^Baseline data from R and B arms (no demographic data specific to no switch (R-R and B-B) and switch (R-B and B-R) arms)

**Table S4. Ethnicity and race of patients for included switch treatment periods**.

| **Characteristic** |  | **STP-01^a^ adalimumab** | **STP-02^b,c^** **adalimumab** | **STP-03^b,c^ adalimumab** | **STP-04^b,c^ adalimumab** | **STP-05^b,c^ adalimumab** | **STP-06 adalimumab** | **STP-07 adalimumab** | **STP-08 adalimumab** | **STP-09 adalimumab** |
| --- | --- | --- | --- | --- | --- | --- | --- | --- | --- | --- |
| **Ethnicity** |  |  |  |  |  |  |  |  |  |  |
| **Hispanic or Latino** | NS, n | 15 | 54 | 54 | 54 | 54 | NR | NR | 18 | 13 |
|  | **NS %** | 13 | 21 | 21 | 21 | 21 | NR | NR | 15 | 10 |
|  | S, n | 8 | 31 | 31 | 31 | 31 | NR | NR | 18 | 10 |
|  | **S %** | 8 | 25 | 25 | 25 | 25 | NR | NR | 15 | 8 |
| **Not Hispanic or Latino** | NS, n | 105 | 201 | 201 | 201 | 201 | NR | NR | 102 | 122 |
|  | **NS %** | 88 | 79 | 79 | 79 | 79 | NR | NR | 85 | 90 |
|  | S, n | 93 | 78 | 78 | 78 | 78 | NR | NR | 100 | 124 |
|  | **S %** | 92 | 62 | 62 | 62 | 62 | NR | NR | 85 | 93 |
| **Race** |  |  |  |  |  |  |  |  |  |  |
| **American Indian or Alaska Native** | NS, n | 5 | 4 | 4 | 4 | 4 | NR | NR | NR | NR |
|  | **NS %** | 4 | 2 | 2 | 2 | 2 | NR | NR | NR | NR |
|  | S, n | 3 | 4 | 4 | 4 | 4 | NR | NR | 0 | NR |
|  | **S %** | 3 | 3 | 3 | 3 | 3 | NR | NR | 0 | NR |
| **Asian** | NS, n | 2 | 3 | 3 | 3 | 3 | NR | 3 | 0 | 8 |
|  | **NS %** | 2 | 1 | 1 | 1 | 1 | NR | 2 | 0 | 6 |
|  | S, n | 7 | 4 | 4 | 4 | 4 | NR | 2 | 2 | 6 |
|  | **S %** | 7 | 3 | 3 | 3 | 3 | NR | 1 | 2 | 5 |
| **Black or African American** | NS, n | 1 | 13 | 13 | 13 | 13 | NR | 1 | 0 | 7 |
|  | **NS %** | 0.8 | 5 | 5 | 5 | 5 | NR | 0.7 | 0 | 5 |
|  | S, n | 0 | 6 | 6 | 6 | 6 | NR | 3 | 2 | 2 |
|  | **S %** | 0 | 5 | 5 | 5 | 5 | NR | 2 | 2 | 2 |
| **Native Hawaiian or Other Pacific Islander** | NS, n | 0 | 1 | 1 | 1 | 1 | NR | NR | 0 | NR |
|  | **NS %** | 0 | 0.4 | 0.4 | 0.4 | 0.4 | NR | NR | 0 | NR |
|  | S, n | 0 | 0 | 0 | 0 | 0 | NR | NR | 1 | NR |
|  | **S %** | 0 | 0 | 0 | 0 | 0 | NR | NR | 1 | NR |
| **White** | NS, n | 110 | 218 | 218 | 218 | 218 | 139 | 141 | 119 | 113 |
|  | **NS %** | 92 | 86 | 86 | 86 | 86 | 87 | 95 | 99 | 84 |
|  | S, n | 90 | 105 | 105 | 105 | 105 | 144 | 140 | 113 | 116 |
|  | **S %** | 89 | 83 | 83 | 83 | 83 | 87 | 96 | 96 | 87 |
| **Other** | NS, n | 2 | 14 | 14 | 14 | 14 | NR | 2 | 1 | 7 |
|  | **NS %** | 2 | 2 | 2 | 2 | 2 | NR | 2 | 1 | 5 |
|  | S, n | 1 | 7 | 7 | 7 | 7 | NR | 1 | 0 | 10 |
|  | **S %** | 1 | 6 | 6 | 6 | 6 | NR | 0.7 | 0 | 8 |

| **Characteristics** |  | **STP-10 adalimumab** | **STP-11 adalimumab** | **STP-12 adalimumab** | **STP-13 adalimumab** | **STP-14 adalimumab** | **STP-15 adalimumab** | **STP-16 adalimumab** | **STP-17 adalimumab** | **STP-18^d^ adalimumab** |
| --- | --- | --- | --- | --- | --- | --- | --- | --- | --- | --- |
| **Ethnicity** |  |  |  |  |  |  |  |  |  |  |
| **Hispanic or Latino** | NS, n | 10 | 17 | 17 | NR | 27 | 1 | 48 | 48 | 23 |
|  | **NS %** | 8 | 13 | 13 | NR | 11.7 | 0.8 | 22.5 | 22.2 | 21.3 |
|  | S, n | 13 | 17 | 17 | NR | 19 | 1 | 23 | 27 | 48 |
|  | **S %** | 11 | 13 | 13 | NR | 8 | 0.8 | 21.3 | 25 | 22.5 |
| **Not Hispanic or Latino** | NS, n | 117 | 119 | 118 | NR | 202 | 128 | 165 | 168 | 85 |
|  | **NS %** | 92 | 88 | 87 | NR | 87.8 | 98.9 | 77.5 | 77.8 | 78.7 |
|  | S, n | 108 | 118 | 119 | NR | 217 | 124 | 85 | 81 | 165 |
|  | **S %** | 89 | 87 | 88 | NR | 91.6 | 99.2 | 78.7 | 75 | 77.5 |
| **Race** |  |  |  |  |  |  |  |  |  |  |
| **American Indian or Alaska Native** | NS, n | NR | 0 | 1 | 0 | NR | 0 | 1 | 1 | 0 |
|  | **NS %** | NR | 0 | 0.7 | 0 | NR | 0 | 1 | 1 | 0 |
|  | S, n | NR | 1 | 0 | 0 | NR | 0 | 0 | 0 | 1 |
|  | **S %** | NR | 0.7 | 0 | 0 | NR | 0 | 0 | 0 | 0.5 |
| **Asian** | NS, n | 6 | 2 | 2 | 3 | 3 | 2 | 0 | 1 | 1 |
|  | **NS %** | 5 | 2 | 2 | 4 | 1 | 2 | 0 | 1 | 0.9 |
|  | S, n | 7 | 2 | 2 | 3 | 0 | 2 | 1 | 0 | 0 |
|  | **S %** | 6 | 2 | 2 | 4 | 0 | 2 | 1 | 0 | 0 |
| **Black or African American** | NS, n | 2 | 1 | 3 | 0 | 8 | 0 | 2 | 1 | 2 |
|  | **NS %** | 2 | 0.7 | 2 | 0 | 4 | 0 | 1 | 1 | 2 |
|  | S, n | 7 | 3 | 1 | 0 | 12 | 0 | 2 | 1 | 2 |
|  | **S %** | 6 | 2 | 0.7 | 0 | 5 | 0 | 2 | 1 | 0.9 |
| **Native Hawaiian or Other Pacific Islander** | NS, n | NR | 0 | 0 | 0 | NR | 0 | NR | NR | NR |
|  | **NS %** | NR | 0 | 0 | 0 | NR | 0 | NR | NR | NR |
|  | S, n | NR | 0 | 0 | 0 | NR | 0 | NR | NR | NR |
|  | **S %** | NR | 0 | 0 | 0 | NR | 0 | NR | NR | NR |
| **White** | NS, n | 109 | 130 | 123 | 74 | 218 | 127 | 185 | 187 | 90 |
|  | **NS %** | 86 | 96 | 91 | 94 | 95 | 98 | 87 | 87 | 83 |
|  | S, n | 100 | 123 | 130 | 69 | 224 | 123 | 90 | 90 | 185 |
|  | **S %** | 83 | 91 | 96 | 90 | 95 | 98 | 83 | 83 | 87 |
| **Other** | NS, n | 10 | 3 | 6 | 0 | 1 | 0 | 25 | 26 | 15 |
|  | **NS %** | 8 | 2 | 4 | 0 | 0.4 | 0 | 12 | 12 | 14 |
|  | S, n | 7 | 6 | 3 | 2 | 1 | 0 | 15 | 17 | 25 |
|  | **S %** | 6 | 4 | 2 | 3 | 0.4 | 0 | 14 | 16 | 12 |

| **Characteristics** |  | **STP-19^e^ adalimumab** | **STP-20 epoetin-alfa** | **STP-21 epoetin-alfa** | **STP-22^f,g^ etanercept** | **STP-23^f,g^ etanercept** | **STP-24 etanercept** | **STP-25 etanercept** | **STP-26 filgrastim** | **STP-27 infliximab** |
| --- | --- | --- | --- | --- | --- | --- | --- | --- | --- | --- |
| **Ethnicity** |  |  |  |  |  |  |  |  |  |  |
| **Hispanic or Latino** | NS, n | 48 | 99 | 90 | NR | NR | NR | NR | 1 | NR |
|  | **NS %** | 22 | 33 | 44 | NR | NR | NR | NR | 2 | NR |
|  | S, n | 27 | 94 | 81 | NR | NR | NR | NR | 0 | NR |
|  | **S %** | 25 | 31 | 38 | NR | NR | NR | NR | 0 | NR |
| **Not Hispanic or Latino** | NS, n | 168 | 204 | 116 | NR | NR | NR | NR | 51 | NR |
|  | **NS %** | 78 | 67 | 56 | NR | NR | NR | NR | 98 | NR |
|  | S, n | 81 | 207 | 130 | NR | NR | NR | NR | 109 | NR |
|  | **S %** | 75 | 69 | 61 | NR | NR | NR | NR | 100 | NR |
| **Race** |  |  |  |  |  |  |  |  |  |  |
| **American Indian or Alaska Native** | NS, n | 1 | NR | 0 | 0 | 0 | NR | 0 | NR | 0 |
|  | **NS %** | 0.5 | NR | 0 | 0 | 0 | NR | 0 | NR | 0 |
|  | S, n | 0 | NR | 0 | NR | NR | NR | NR | NR | 0 |
|  | **S %** | 0 | NR | 0 | NR | NR | NR | NR | NR | 0 |
| **Asian** | NS, n | 1 | NR | 9 | 1 | 0 | NR | 0 | NR | 13 |
|  | **NS %** | 0.5 | NR | 4 | 0.7 | 0 | NR | 0 | NR | 13 |
|  | S, n | 0 | NR | 11 | 0 | 0 | NR | NR | NR | 7 |
|  | **S %** | 0 | NR | 5 | 0 | 0 | NR | NR | NR | 8 |
| **Black or African American** | NS, n | 1 | 125 | 47 | 0 | 0 | NR | 0 | NR | 0 |
|  | **NS %** | 0.5 | 41 | 23 | 0 | 0 | NR | 0 | NR | 0 |
|  | S, n | 1 | 146 | 60 | NR | NR | NR | NR | NR | 0 |
|  | **S %** | 0.9 | 49 | 28 | NR | NR | NR | NR | NR | 0 |
| **Native Hawaiian or Other Pacific Islander** | NS, n | NR | NR | 0 | 0 | 0 | NR | 0 | NR | 0 |
|  | **NS %** | NR | NR | 0 | 0 | 0 | NR | 0 | NR | 0 |
|  | S, n | NR | NR | 1 | NR | NR | NR | NR | NR | 0 |
|  | **S %** | NR | NR | 0.5 | NR | NR | NR | NR | NR | 0 |
| **White** | NS, n | 187 | 150 | 146 | 150 | 150 | 169 | 126 | NR | 88 |
|  | **NS %** | 87 | 49 | 70.9 | 99 | 100 | 97 | 100 | NR | 87 |
|  | S, n | 90 | 141 | 137 | 194 | 194 | 164 | 118 | NR | 87 |
|  | **S %** | 83 | 47 | 65 | 99 | 99 | 99 | 99 | NR | 95 |
| **Other** | NS, *n* | 26 | 29 | 4 | 0 | 0 | NR | 0 | NR | 0 |
|  | **NS %** | 12 | 10 | 2 | 0 | 0 | NR | 0 | NR | 0 |
|  | S, *n* | 17 | 14 | 3 | 2 | 2 | NR | NR | NR | 0 |
|  | **S %** | 16 | 4 | 1 | 1 | 1 | NR | NR | NR | 0 |

| **Characteristics** |  | **STP-28 infliximab** | **STP-29 infliximab** | **STP-30 infliximab** | **STP-31 infliximab** | **STP-32 infliximab** | **STP-33^g^ infliximab** | **STP-34^g^ infliximab** | **STP-35 infliximab** | **STP-36 infliximab** |
| --- | --- | --- | --- | --- | --- | --- | --- | --- | --- | --- |
| **Ethnicity** |  |  |  |  |  |  |  |  |  |  |
| **Hispanic or Latino** | NS, n | 9 | NR | NR | NR | NR | NR | NR | NR | 15 |
|  | **NS %** | 7 | NR | NR | NR | NR | NR | NR | NR | 12 |
|  | S, n | 4 | NR | NR | NR | NR | NR | NR | NR | 10 |
|  | **S %** | 3.4 | NR | NR | NR | NR | NR | NR | NR | 8 |
| **Not Hispanic or Latino** | NS, n | 112 | NR | NR | NR | NR | NR | NR | NR | 111 |
|  | **NS %** | 93 | NR | NR | NR | NR | NR | NR | NR | 88 |
|  | S, n | 115 | NR | NR | NR | NR | NR | NR | NR | 116 |
|  | **S %** | 97 | NR | NR | NR | NR | NR | NR | NR | 92 |
| **Race** |  |  |  |  |  |  |  |  |  |  |
| **American Indian or Alaska Native** | NS, n | 0 | NR | 0 | NR | NR | NR | NR | NR | NR |
|  | **NS %** | 0 | NR | 0 | NR | NR | NR | NR | NR | NR |
|  | S, n | 0 | NR | 0 | NR | NR | NR | NR | NR | NR |
|  | **S %** | 0 | NR | 0 | NR | NR | NR | NR | NR | NR |
| **Asian** | NS, n | 0 | 17 | 10 | NR | NR | R:29  B: 25 | R:29  B: 25 | 23 | 16 |
|  | **NS %** | 0 | 11 | 11 | NR | NR | R: 27  B: 23 | R: 27  B: 23 | 16 | 13 |
|  | S, n | 0 | 10 | 11 | NR | NR | NR | NR | 20 | 18 |
|  | **S %** | 0 | 7 | 13 | NR | NR | NR | NR | 14 | 14 |
| **Black or African American** | NS, n | 4 | 1 | 0 | NR | NR | NR | NR | 4 | NR |
|  | **NS %** | 3 | 0.6 | 0 | NR | NR | NR | NR | 3 | NR |
|  | S, n | 6 | 1 | 0 | NR | NR | NR | NR | 4 | NR |
|  | **S %** | 5 | 0.7 | 0 | NR | NR | NR | NR | 2.8 | NR |
| **Native Hawaiian or Other Pacific Islander** | NS, n | 0 | NR | 0 | NR | NR | NR | NR | NR | NR |
|  | **NS %** | 0 | NR | 0 | NR | NR | NR | NR | NR | NR |
|  | S, n | 0 | NR | 0 | NR | NR | NR | NR | NR | NR |
|  | **S %** | 0 | NR | 0 | NR | NR | NR | NR | NR | NR |
| **White** | NS, n | 117 | 119 | 70 | NR | NR | R:79  B: 86 | R:79  B: 86 | 104 | 97 |
|  | **NS %** | 97 | 75 | 80 | NR | NR | R: 72  B: 77 | R: 72  B: 77 | 72.7 | 77 |
|  | S, n | 113 | 105 | 61 | NR | NR | NR | NR | 109 | 93 |
|  | **S %** | 95 | 73 | 71 | NR | NR | NR | NR | 76 | 74 |
| **Other** | NS, n | 0 | 21 | 8 | NR | NR | R:1  B: 0 | R:1  B: 0 | 12 | NR |
|  | **NS %** | 0 | 13 | 9 | NR | NR | R: 1  B: 0 | R: 1  B: 0 | 8 | NR |
|  | S, n | 0 | 28 | 14 | NR | NR | NR | NR | 10 | NR |
|  | **S %** | 0 | 19 | 16 | NR | NR | NR | NR | 7 | NR |

| **Characteristics** | **Arm** | **STP-37 insulin-glargine** | **STP-38^b^ rituximab** | **STP-39 rituximab** | **STP-40 rituximab** | **STP-41^b^ rituximab** | **STP-42^b,c^ rituximab** | **STP-43^b^** **rituximab** | **STP-44 trastuzumab** |
| --- | --- | --- | --- | --- | --- | --- | --- | --- | --- |
| **Ethnicity** |  |  |  |  |  |  |  |  |  |
| **Hispanic or Latino** | NS, n | NR | NR | NR | NR | 10 | NR | NR | NR |
|  | **NS %** | NR | NR | NR | NR | 10 | NR | NR | NR |
|  | S, n | NR | NR | NR | NR | 11 | NR | NR | NR |
|  | **S %** | NR | NR | NR | NR | 11 | NR | NR | NR |
| **Not Hispanic or Latino** | NS, n | NR | NR | NR | NR | 94 | NR | NR | NR |
|  | **NS %** | NR | NR | NR | NR | 90 | NR | NR | NR |
|  | S, n | NR | NR | NR | NR | 92 | NR | NR | NR |
|  | **S %** | NR | NR | NR | NR | 89 | NR | NR | NR |
| **Race** |  |  |  |  |  |  |  |  |  |
| **American Indian or Alaska Native** | NS, n | NR | NR | NR | NR | 0 | NR | NR | 0 |
|  | **NS %** | NR | NR | NR | NR | 0 | NR | NR | 0 |
|  | S, n | NR | NR | NR | NR | 0 | NR | NR | 0 |
|  | **S %** | NR | NR | NR | NR | 0 | NR | NR | 0 |
| **Asian** | NS, n | 0 | 4 | NR | 47 | 2 | 1 | 0 | 2 |
|  | **NS %** | 0 | 6 | NR | 36 | 2 | 0.7 | 0 | 1.2 |
|  | S, n | 2 | 5 | NR | 49 | 1 | NR | 0 | 1 |
|  | **S %** | 3 | 5 | NR | 38 | 1 | NR | 0 | 0.6 |
| **Black or African American** | NS, n | 2 | NR | NR | NR | 3 | 11 | 6 | 0 |
|  | **NS %** | 3 | NR | NR | NR | 3 | 8 | 10 | 0 |
|  | S, n | 2 | NR | NR | NR | 10 | NR | 5 | 2 |
|  | **S %** | 3.1 | NR | NR | NR | 10 | NR | 8 | 1 |
| **Native Hawaiian or Other Pacific Islander** | NS, n | NR | NR | NR | NR | NR | NR | NR | NR |
|  | **NS %** | NR | NR | NR | NR | NR | NR | NR | NR |
|  | S, n | NR | NR | NR | NR | NR | NR | NR | NR |
|  | **S %** | NR | NR | NR | NR | NR | NR | NR | NR |
| **White** | NS, n | 61 | 41 | 23 | 77 | 99 | 115 | 48 | 158 |
|  | **NS %** | 97 | 64 | 60.5 | 59 | 95 | 78 | 76 | 92 |
|  | S, n | 59 | 70 | 11 | 75 | 91 | NR | 47 | 158 |
|  | **S %** | 92 | 64 | 55 | 59 | 88 | NR | 75 | 92 |
| **Other** | NS, n | 0 | 19 | NR | 6 | 0 | 20 | 9 | 11 |
|  | **NS %** | 0 | 30 | NR | 5 | 0 | 14 | 14 | 6 |
|  | S, n | 1 | 34 | NR | 4 | 1 | NR | 11 | 10 |
|  | **S %** | 2 | 31 | NR | 3 | 1 | NR | 18 | 6 |

B, Biosimilar Product; NR, Not Reported; R, Reference Biologic

^a^Baseline data for No Switch arm is from R at time of entry into study.

^b^Sufficient data included in the submission to justify combining E.U. and U.S. Reference Biologic arms.

^c^Baseline data at time of entry into study

^d^Baseline data at time of entry into STP-16

^e^Baseline data at time of entry into STP-17

^f^Baseline data for switch arm is pooled for both switch arms (R-B and B-R).

^g^Baseline data from R and B arms (no demographic data specific to no switch (R-R and B-B) and switch (R-B and B-R) arms)

**Table S5. Exposure to study drug.**

| **Duration of Exposure (days)** | | | |
| --- | --- | --- | --- |
| **Switch Treatment Period** | **Reference Product** | **No Switch**  **(Mean (SD); Min, Max)** | **Switch**  **(Mean (SD); Min, Max)** |
| STP-01^a^ | adalimumab | 295 (117); 357; 7, 364 | 344 (47); 357; 117, 364 |
| STP-02^b,c^ | adalimumab | 93; (17); 1, 227 | 96; (24);1, 230 |
| STP-03^b,c^ | adalimumab | 93; (17); 1, 227 | 96; (24);1, 230 |
| STP-04^b,c^ | adalimumab | 93; (17); 1, 227 | 96; (24);1, 230 |
| STP-05^b,c^ | adalimumab | 93; (17); 1, 227 | 96; (24);1, 230 |
| STP-06 | adalimumab | NR (NR); 1, 353 | NR (NR); 1, 345 |
| STP-07 | adalimumab | 311 (38); NR, NR | 311 (35); NR, NR |
| STP-08 | adalimumab | 311 (50); NR, NR | 319 (42); NR, NR |
| STP-09 | adalimumab | NR (NR); 169; 1, 184 | NR (NR); 169; 1, 183 |
| STP-10 | adalimumab | NR (NR); 1, 183)  (median=169) | NR (NR); 1, 184)  (median=169) |
| STP-11 | adalimumab | 309 (69); 336; 14, 350 | 292 (82); 336; 7, 371 |
| STP-12 | adalimumab | 292 (82); 336; 7, 371 | 309 (69); 336; 14, 350 |
| STP-13 | adalimumab | 209 (51); NR, NR | 211 (46); NR, NR |
| STP-14 | adalimumab | NR (NR); NR, NR | NR (NR); NR, NR |
| STP-15 | adalimumab | 348 (22); 169, 364 | 343 (33); 183, 362 |
| STP-16 | adalimumab | 318 (59); 14, 351 | 317 (63); 14, 371 |
| STP-17 | adalimumab | 319 (60); 14, 348 | 314 (66); 14, 350 |
| STP-18^d^ | adalimumab | 317 (63); 14, 371 | 318 (59); 14, 351 |
| STP-19^e^ | adalimumab | 319 (60); 14, 348 | 314 (66); 14, 350 |
| STP-20 | epoetin-alfa | 137 (50); 1, 178 | 139 (46); 1, 174 |
| STP-21 | epoetin-alfa | 139 (49); NR, NR | 136 (46); NR, NR |
| STP-22^f^ | etanercept | 117 (15); 1, 134 | 117 (15); 8, 169 |
| STP-23^f^ | etanercept | 117 (16); 8, 134 | 117 (15); 8, 169 |
| STP-24 | etanercept | NR (NR); 162; 8, 174 | NR (NR) 162; 1, 169 |
| STP-25 | etanercept | 683 (NR); NR, NR | 683 (NR); NR, NR |
| STP-26 | filgrastim | NR (NR); NR, NR | NR (NR); NR, NR |
| STP-27 | infliximab | 110 (16); 1, 130 | 111 (13); 51, 128 |
| STP-28 | infliximab | 313 (47); 161, 358 | 315 (47); 182, 358 |
| STP-29 | infliximab | NR (NR); NR, NR  (total dose of B administered during study (mg) 1234 (393); 0, 2361) | NR (NR); NR, NR  (total dose of B administered during study (mg) 1234 (393); 0, 2361) |
| STP-30 | infliximab | NR (NR); NR, NR  (total dose of B administered during study (mg) 2263 (576); 509, 3599) | NR (NR); NR, NR  (total dose of B administered during study (mg) 2277 (581); 380, 3715) |
| STP-31 | infliximab | NR (NR); NR, NR | NR (NR); NR, NR |
| STP-32 | infliximab | NR (NR); NR, NR | NR (NR); NR, NR |
| STP-33 | infliximab | NR (NR); NR, NR | NR (NR); NR, NR |
| STP-34 | infliximab | NR (NR); NR, NR | NR (NR); NR, NR |
| STP-35 | infliximab | NR (NR); 113; 1, 148 | NR (NR); 113; 1, 142 |
| STP-36 | infliximab | NR (NR); NR, NR  (mean dose administered (mg) 790±319) | NR (NR); NR, NR  (mean dose administered (mg) 762±369) |
| STP-37 | insulin-glargine | 246 (31); 93, 282 | 251 (17); 144, 274 |
| STP-38^b^ | rituximab | NR (NR); NR, NR  (total dose administered (mg) 2000 (0), 2000, 2000) | NR (NR); NR, NR  (total dose administered (mg) 1991 (96), 1000, 2000) |
| STP-39 | rituximab | NR (NR); NR, NR  (total Dose total dose administered (mg) 2026 (367); 1000, 4000) | NR (NR); NR, NR  (total Dose total dose administered (mg) 2000 (0); 2000, 2000) |
| STP-40 | rituximab | NR (NR); NR, NR | NR (NR); NR, NR |
| STP-41^b^ | rituximab | 323 (49); NR, NR | 320 (67); NR, NR |
| STP-42^b,c^ | rituximab | NR (NR); NR, NR  (actual dose 1000 mg on Day 1 and Day 15 (0); 1000, 1000) | NR (NR); NR, NR  (actual dose 1000 mg on Day 1 and Day 15 (0); 1000, 1000 |
| STP-43^b^ | rituximab | NR (NR); NR, NR  (actual dose 1000 mg on Day 1 and Day 15 (0); 1000, 1000 | NR (NR); NR, NR  (actual dose 1000 mg on Day 1 and Day 15 (0); 1000, 1000 |
| STP-44 | trastuzumab | NR (NR); NR, NR  (weight based cumulative dose administered (mg/kg): 91 (25); 8, 108) | NR (NR); NR, NR  (weight based cumulative dose administered (mg/kg) 98 (12); 34, 108) |

B, Biosimilar Product; NR, Not Reported; R, Reference Biologic

^a^Baseline data for No Switch arm is from R at time of entry into study.

^b^Sufficient data included in the submission to justify combining E.U. and U.S. Reference Biologic arms.

^c^Baseline data at time of entry into study

^d^Baseline data at time of entry into STP-16

^e^Baseline data at time of entry into STP-17

^f^Baseline data for switch arm is pooled for both switch arms (R-B and B-R).

**Table S6. Deaths, severe adverse events, and discontinuations.**

|  | | | | **Deaths** | | **Serious Adverse Events (SAE)** | | **Discontinuations** | |
| --- | --- | --- | --- | --- | --- | --- | --- | --- | --- |
| **Switch Treatment Period** | **Reference Product** | **No Switch  (N)** | **Switch (N)** | **No Switch n (%)** | **Switch n (%)** | **No Switch n (%)** | **Switch n (%)** | **No Switch n (%)** | **Switch n (%)** |
| STP-01 | adalimumab | 101 | 101 | 1 (1) | 0 (0) | 3 (3) | 4 (4) | 6 (6) | 4 (4) |
| STP-02^a^ | adalimumab | 127 | 63 | 0 (0) | 0 (0) | 2 (2) | 1 (2) | 2 (2) | 1 (2) |
| STP-03^a^ | adalimumab | 126 | 63 | 1 (1) | 0 (0) | 1 (1) | 1 (2) | 2 (2) | 0 (0) |
| STP-04^a^ | adalimumab | 109 | 56 | 0 (0) | 0 (0) | 0 (0) | 1 (2) | 0 (0) | 0 (0) |
| STP-05^a^ | adalimumab | 106 | 52 | 1 (1) | 0 (0) | 0 (0) | 0 (0) | 0 (0) | 0 (0) |
| STP-06 | adalimumab | 159 | 166 | 0 (0) | 0 (0) | 7 (4) | 10 (6) | 9 (5) | 3 (2) |
| STP-07 | adalimumab | 148 | 146 | 0 (0) | 0 (0) | 5 (3) | 2 (1) | 1 (1) | 2 (1) |
| STP-08 | adalimumab | 120 | 118 | 0 (0) | 0 (0) | 4 (3) | 5 (4) | 2 (2) | 1 (1) |
| STP-09 | adalimumab | 135 | 133 | 0 (0) | 0 (0) | 6 (4) | 3 (2) | 8 (6) | 2 (2) |
| STP-10 | adalimumab | 127 | 120 | 0 (0) | 1 (0) | 3 (2) | 9 (8) | 2 (2) | 3 (3) |
| STP-11 | adalimumab | 130 | 126 | 0 (0) | 0 (0) | 1 (1) | 3 (2) | 1 (1) | 2 (2) |
| STP-12 | adalimumab | 113 | 126 | 0 (0) | 0 (0) | 3 (3) | 0 (0) | 2 (2) | 0 (0) |
| STP-13 | adalimumab | 79 | 77 | 0 (0) | 0 (0) | 4 (5) | 4 (5) | 1 (1) | 2 (3) |
| STP-14 | adalimumab | 230 | 237 | 0 (0) | 0 (0) | 21 (9) | 25 (11) | 4 (2) | 6 (3) |
| STP-15 | adalimumab | 127 | 125 | 0 (0) | 0 (0) | 4 (3) | 4 (3) | 3 (2) | 2 (2) |
| STP-16 | adalimumab | 213 | 108 | 1 (1) | 1 (1) | 7 (3) | 5 (5) | 11 (5) | 4 (4) |
| STP-17 | adalimumab | 216 | 108 | 0 (0) | 0 (0) | 5 (2) | 7 (7) | 10 (5) | 0 (0) |
| STP-18 | adalimumab | 93 | 190 | 1 (1) | 1 (1) | 6 (7) | 11 (6) | 6 (7) | 10 (5) |
| STP-19 | adalimumab | 189 | 100 | 0 (0) | 0 (0) | 8 (4) | 8 (8) | 4 (2) | 5 (5) |
| STP-20 | epoetin alfa | 304 | 301 | 6 (2) | 5 (2) | 82 (27) | 75 (25) | 8 (3) | 8 (3) |
| STP-21 | epoetin alfa | 206 | 212 | 9 (4) | 10 (5) | 64 (31) | 66 (31) | 9 (4) | 13 (6) |
| STP-22 | etanercept | 151 | 96 | 0 (0) | 0 (0) | 2 (1) | 3 (3) | 2 (1) | 5 (5) |
| STP-23 | etanercept | 150 | 100 | 0 (0) | 0 (0) | 1 (1) | 3 (3) | 1 (1) | 1 (1) |
| STP-24 | etanercept | 175 | 166 | 0 (0) | 0 (0) | 4 (2) | 4 (2) | 4 (2) | 4 (2) |
| STP-25 | etanercept | 126 | 119 | 1 (1) | 0 (0) | 6 (5) | 2 (2) | 4 (3) | 2 (2) |
| STP-26 | filgrastim | 52 | 109 | 0 (0) | 0 (0) | 1 (2) | 5 (5) | 0 (0) | 0 (0) |
| STP-27 | infliximab | 101 | 94 | 0 (0) | 0 (0) | 3 (3) | 6 (6) | 3 (3) | 3 (3) |
| STP-28 | infliximab | 121 | 119 | 0 (0) | 0 (0) | 3 (3) | 1 (1) | 4 (3) | 3 (3) |
| STP-29 | infliximab | 158 | 144 | 1 (1) | 0 (0) | 12 (8) | 19 (9) | 8 (6) | 16 (10) |
| STP-30 | infliximab | 90 | 84 | 0 (0) | 0 (0) | 4 (4) | 4 (4) | 3 (3) | 4 (5) |
| STP-31 | infliximab | 241 | 241 | 0 (0) | 0 (0) | 24 (10) | 21 (9) | 9 (4) | 8 (3) |
| STP-32 | infliximab | 197 | 183 | 0 (0) | 0 (0) | 14 (7) | 8 (4) | 2 (1) | 1 (1) |
| STP-33 | infliximab | 54 | 55 | 0 (0) | 0 (0) | 4 (7) | 7 (13) | 3 (6) | 3 (5) |
| STP-34 | infliximab | 56 | 55 | 0 (0) | 0 (0) | 4 (7) | 4 (7) | 3 (5) | 3 (5) |
| STP-35 | infliximab | 143 | 143 | 0 (0) | 0 (0) | 11 (8) | 4 (3) | 10 (7) | 7 (5) |
| STP-36 | infliximab | 126 | 126 | 0 (0) | 0 (0) | 6 (5) | 3 (2) | 2 (2) | 2 (2) |
| STP-37 | insulin glargine | 63 | 64 | 1 (2) | 0 (0) | 5 (8) | 3 (5) | 1 (2) | 1 (2) |
| STP-38^a^ | rituximab | 64 | 109 | 0 (0) | 0 (0) | 0 (0) | 1 (1) | 0 (0) | 0 (0) |
| STP-39 | rituximab | 38 | 20 | 0 (0) | 0 (0) | 2 (3) | 1 (5) | 0 (0) | 0 (0) |
| STP-40 | rituximab | 110 | 103 | 0 (0) | 0 (0) | 71 (69) | 68 (62) | 2 (2) | 0 (0) |
| STP-41^a^ | rituximab | 104 | 103 | 0 (0) | 0 (0) | 8 (8) | 8 (8) | 2 (2) | 7 (7) |
| STP-42^a^ | rituximab | 62 | 63 | 0 (0) | 0 (0) | 3 (5) | 4 (6) | 2 (3) | 2 (3) |
| STP-43^a^ | rituximab | 59 | 57 | 0 (0) | 0 (0) | 5 (1) | 2 (4) | 1 (2) | 0 (0) |
| STP-44 | trastuzumab | 171 | 171 | 0 (0) | 3 (2) | 9 (5) | 11 (6) | 3 (2) | 2 (1) |

^a^Sufficient data included in the submission to justify combining E.U. and U.S. Reference Biologic arms.

**Table S7. Antidrug antibody and neutralizing antibody data.**

Collected after completion of switch treatment period. Antibody positive patients expressed as a percentage of total patients.

| **Switch Treatment Period** | **Reference Product** | **Antidrug Antibody (%)** | | | | **Neutralizing Antibody (%)** | | | |
| --- | --- | --- | --- | --- | --- | --- | --- | --- | --- |
|  |  | **Pre** | | **Post** | | **Pre** | | **Post** | |
|  |  | **No Switch** | **Switch** | **No Switch** | **Switch** | **No Switch** | **Switch** | **No Switch** | **Switch** |
| STP-01 | adalimumab | NR | NR | 94 | 94 | NR | NR | 53 | 62 |
| STP-02^a^ | adalimumab | 22 | 21 | 21 | 32 | 21 | 21 | 20 | 32 |
| STP-03^a^ | adalimumab | 20 | 29 | 15 | 26 | 19 | 27 | 15 | 26 |
| STP-04^a^ | adalimumab | 21 | 26 | 19 | 28 | 20 | 26 | 18 | 28 |
| STP-05^a^ | adalimumab | 15 | 32 | 17 | 33 | 15 | 32 | 17 | 33 |
| STP-06 | adalimumab | NR | NR | 24 | 26 | NR | NR | 17 | 21 |
| STP-07 | adalimumab | 50 | 45 | 47 | 38 | 24 | 15 | 13 | 16 |
| STP-08 | adalimumab | 86 | 88 | 87 | 84 | 41 | 44 | 36 | 38 |
| STP-09 | adalimumab | 47 | 45 | 43 | 41 | 11 | 10 | 8 | 9 |
| STP-10 | adalimumab | 43 | 48 | 47 | 54 | 9 | 8 | 8 | 25 |
| STP-11 | adalimumab | 94 | 94 | 96 | 95 | 33 | 33 | 40 | 41 |
| STP-12 | adalimumab | 95 | 96 | 97 | 98 | 41 | 40 | 56 | 57 |
| STP-13 | adalimumab | 54 | 62 | 43 | 55 | 11 | 10 | 13 | 21 |
| STP-14 | adalimumab | NR | NR | 22 | 15 | NR | NR | 9 | 5 |
| STP-15 | adalimumab | 28 | 30 | 33 | 31 | 14 | 14 | 22 | 18 |
| STP-16 | adalimumab | 58 | 62 | 52 | 45 | 58 | 62 | 51 | 45 |
| STP-17 | adalimumab | 62 | 64 | 52 | 61 | 62 | 62 | 52 | 60 |
| STP-18 | adalimumab | 45 | 52 | 48 | 43 | 45 | 51 | 47 | 42 |
| STP-19 | adalimumab | 52 | 61 | 51 | 54 | 52 | 60 | 51 | 54 |
| STP-20 | epoetin-alfa | NR | NR | NR | NR | 0 | 0 | 0 | 0 |
| STP-21 | epoetin-alfa | NR | NR | NR | NR | NR | NR | NR | NR |
| STP-22 | etanercept | 0 | 0 | 0 | 0 | 0 | 0 | 0 | 0 |
| STP-23 | etanercept | 0 | 0 | 0 | 0 | 0 | 0 | 0 | 0 |
| STP-24 | etanercept | 0 | 0 | 4 | 2 | 0 | 0 | 0 | 0 |
| STP-25 | etanercept | 1 | 0 | 1 | 1 | 0 | 0 | 0 | 0 |
| STP-26 | filgrastim | 0 | 0 | 0 | 0 | 0 | 0 | 0 | 0 |
| STP-27 | infliximab | 44 | 33 | 51 | 63 | NR | NR | 45 | 40 |
| STP-28 | infliximab | 57 | 56 | 27 | 24 | 21 | 16 | 7 | 8 |
| STP-29 | infliximab | NR | NR | 40 | 45 | 49 | 45 | 40 | 45 |
| STP-30 | infliximab | 22 | 26 | 23 | 27 | 22 | 26 | 23 | 27 |
| STP-31 | infliximab | NR | NR | NR | NR | NR | NR | NR | NR |
| STP-32 | infliximab | NR | NR | NR | NR | NR | NR | NR | NR |
| STP-33 | infliximab | NR | NR | 39 | 55 | NR | NR | NR | NR |
| STP-34 | infliximab | NR | NR | 39 | 33 | NR | NR | NR | NR |
| STP-35 | infliximab | 54 | 46 | 60 | 58 | 45 | 38 | 51 | 45 |
| STP-36 | infliximab | 53 | 48 | 49 | 44 | 39 | 36 | 41 | 33 |
| STP-37 | insulin-glargine | 65 | 75 | 54 | 70 | 67 | 73 | 56 | 67 |
| STP-38^a^ | rituximab | 14 | 21 | 3 | 10 | 0 | 1 | 0 | 0 |
| STP-39 | rituximab | NR | NR | 13 | 15 | NR | NR | 3 | 0 |
| STP-40 | rituximab | NR | NR | 0 | 0 | NR | NR | 0 | 0 |
| STP-41^a^ | rituximab | 11 | 19 | 14 | 21 | 2 | 8 | 4 | 10 |
| STP-42^a^ | rituximab | NR | NR | NR | NR | NR | NR | NR | NR |
| STP-43^a^ | rituximab | NR | NR | NR | NR | NR | NR | NR | NR |
| STP-44 | trastuzumab | 1 | 2 | 0 | 1 | 0 | 0 | 0 | 0 |

NR, Not Reported

^a^Sufficient data included in the submission to justify combining E.U. and U.S. Reference Biologic arms.

**Table S8. Hypersensitivity, infusion reactions, and injection site reactions.**

| **Switch Treatment Period** | **Reference Product** | **No Switch  (*n* patients with AE of interest/N arm)** | **No Switch (%)** | **Switch  (*n* patients with AE of interest/N arm)** | **Switch  (%)** |  |
| --- | --- | --- | --- | --- | --- | --- |
| **Hypersensitivity (includes Infusion Reactions)** | | | | | | |
| STP-07 | adalimumab | 2/148 | 1 | 3/146 | 2 |  |
| STP-08 | adalimumab | 2/120 | 2 | 4/118 | 3 |  |
| STP-09 | adalimumab | 6/135 | 4 | 5/133 | 4 |  |
| STP-11 | adalimumab | 0/130 | 0 | 1/126 | 1 |  |
| STP-13 | adalimumab | 2/79 | 2 | 3/77 | 4 |  |
| STP-16 | adalimumab | 10/213 | 5 | 2/108 | 2 |  |
| STP-17 | adalimumab | 8/216 | 4 | 8/108 | 7 |  |
| STP-18 | adalimumab | 1/93 | 1 | 8/190 | 4 |  |
| STP-19 | adalimumab | 4/189 | 2 | 6/100 | 6 |  |
| STP-22 | etanercept | 1/151 | 1 | 0/96 | 0 |  |
| STP-23 | etanercept | 0/150 | 0 | 0/100 | 0 |  |
| STP-25 | etanercept | 1/126 | 1 | 0/119 | 0 |  |
| STP-27 | infliximab | 2/101 | 2 | 4/94 | 3 |  |
| STP-28 | infliximab | 3/121 | 3 | 0/119 | 0 |  |
| STP-35 | infliximab | 24/143 | 17 | 14/143 | 10 |  |
| STP-38 | rituximab | 3/64 | 5 | 4/109 | 4 |  |
| STP-39 | rituximab | 1/38 | 3 | 1/20 | 5 |  |
| STP-40 | rituximab | 9/104 | 9 | 16/103 | 16 |  |
| STP-41 | rituximab | 1/62 | 2 | 1/63 | 2 |  |
| STP-42^a^ | rituximab | 2/60 | 3 | 1/59 | 2 |  |
| STP-43 | trastuzumab | 21/171 | 12 | 28/171 | 16 |  |
| **Hypersensitivity Risk Difference Summary Statistics** | | | | |  |  |
| Mean 0.57  Median 1  Range -7, 7  95% CI (-0.69, 1.84) | |  | | | |  |
| **Injection Site Reactions** | | | | | | |
| STP-07 | adalimumab | 2/148 | 1 | 0/146 | 0 |  |
| STP-08 | adalimumab | 5/120 | 4 | 3/118 | 3 |  |
| STP-09 | adalimumab | 0/135 | 0 | 1/133 | 1 |  |
| STP-11 | adalimumab | 2/130 | 2 | 0/126 | 0 |  |
| STP-16 | adalimumab | 3/213 | 1 | 1/108 | 1 |  |
| STP-17 | adalimumab | 3/216 | 1 | 2/108 | 2 |  |
| STP-18 | adalimumab | 0/190 | 0 | 1/93 | 1 |  |
| STP-19 | adalimumab | 3/189 | 2 | 2/100 | 2 |  |
| STP-20 | epoetin-alfa | 8/304 | 3 | 3/301 | 1 |  |
| STP-22 | etanercept | 7/151 | 5 | 4/96 | 4 |  |
| STP-23 | etanercept | 6/150 | 4 | 5/100 | 5 |  |
| STP-25 | etanercept | 0/126 | 0 | 0/119 | 0 |  |
| **Injection Site Reactions Risk Difference Summary Statistics** | | | | | |  |
| Mean -0.25  Median 0  Range -2, 1  95% CI (-0.89, 0.39) | |  | | | |  |

AE, Adverse Event; CI, Confidence Interval
^a^Denominators different from Table 1 as Table 1 data is from publication (Cohen, 2020) and these are from FDA Reviews

**Table S9. Antidrug antibody and neutralizing antibody status in patients with hypersensitivity, infusion reactions, and injection site reactions.**

|  |  | **No Switch** | | | | | | **Switch** | | | | | | | | |
| --- | --- | --- | --- | --- | --- | --- | --- | --- | --- | --- | --- | --- | --- | --- | --- | --- |
| **Switch Treatment Period** | **Reference Product** | **ADA+ (n with AE/**  **n ADA+)** | **ADA- (n with AE/**  **n ADA-)** | **NAb+  (n with AE/**  **n NAb+)** | **ADA+ (%)** | **ADA- (%)** | **NAb+ (%)** | **ADA+ (n with AE/**  **n ADA+)** | **ADA- (n with AE/**  **n ADA-)** | **NAb+  (n with AE/**  **n NAB+)** | **ADA+ (%)** | **ADA- (%)** | | **NAb+ (%)** | |  |
| **Hypersensitivity (includes Infusion Reactions)** | | | | | | | | | |  |  | |  | |  | |
| STP-11 | adalimumab | 0/121 | 0/9 | 0/38 | 0 | 0 | 0 | 0/119 | 1/6 | 0/44 | 0 | 17 | | 0 | |  |
| **Injection Site Reactions** | | | | | | | | | | | | | | | | |
| STP-11 | adalimumab | 2/121 | 0/9 | 0/38 | 2 | 0 | 0 | 0 | 0 | 0 | 0 | 0 | | 0 | |  |
| STP-16 | adalimumab | 0 | 3/102 | 1/116 | 0 | 3 | 1 | 0 | 1/57 | 0 | 0 | 2 | | 0 | |  |
| STP-17 | adalimumab | 0 | 3/105 | 0 | 0 | 3 | 0 | 0 | 0 | 2/67 | 3 | 0 | | 3 | |  |
| STP-18 | adalimumab | 0 | 1/49 | 0 | 0 | 2 | 0 | 0 | 0 | 0 | 0 | 0 | | 0 | |  |
| STP-19 | adalimumab | 1/97 | 2/83 | 1/97 | 1 | 2 | 1 | 1/52 | 1/41 | 1/52 | 2 | 2 | | 2 | |  |

ADA, Anti-Drug Antibody; AE, Adverse Event; NAb, Neutralizing Antibody

**Table S10. Un-adjusted and adjusted odds ratio of death by individual factor and biosimilar drug.**

Exploratory Logistic regression analysis was performed to evaluate the impact of heterogeneity of different studies on study conclusions. Adjusted Odds Ratios (OR) from multiple logistic regression models were adjusted for the biosimilar drugs assessed and each individual factor (study design, patient population, time on study drug prior to switching, duration of switch, number of switches during STP, mean age, percent sex, and mean BMI).

| **Factor** | **OR (S vs NS) (90% CI)** | **P value** |
| --- | --- | --- |
| Unadjusted (S vs NS) | 1.003 (0.610, 1.650) | 0.9918 |
| Adjusted for^a^: |  |  |
| Study Design^b^ | 0.954 (0.576, 1.579) | 0.8770 |
| Patient Population^c^ | 0.969 (0.586, 1.604) | 0.9192 |
| Duration on Study Drug before Switch (Days) | 0.845 (0.488, 1.465) | 0.6154 |
| STP Duration (Weeks) | 0.955 (0.577, 1.580) | 0.8799 |
| Number of switches during STP | 0.957 (0.579, 1.583) | 0.8857 |
| Mean Age | 0.873 (0.519, 1.471) | 0.6691 |
| Gender (%) | 0.958 (0.579, 1.585) | 0.8891 |
| Mean BMI | 0.728 (0.409, 1.293) | 0.3633 |

^a^Adjusted odds ratios are from multiple logistic regression models adjusted by each individual factor and the biosimilar drugs (i.e., BLA study number).

^b^Study Design: Randomized Double Blind, Randomized Open Label, Open Label Extension.

^c^Patient Population: Ankylosing Spondylitis, Breast Cancer, Crohn Disease, Chronic Kidney Disease, Follicular Lymphoma, Psoriatic Arthritis, Plaque Psoriasis, Rheumatoid Arthritis, Spondylarthritis, Type 1 Diabetes Mellitus, Ulcerative Colitis

**Table S11. Un-adjusted and adjusted odds ratio of SAE by individual factor and biosimilar drug.**

Exploratory Logistic regression analysis was performed to evaluate the impact of heterogeneity of different studies on study conclusions. Adjusted Odds Ratios (OR) from multiple logistic regression models were adjusted for the biosimilar drugs assessed and each individual factor (study design, patient population, time on study drug prior to switching, duration of switch, number of switches during STP, mean age, percent sex, and mean BMI).

| **Factor** | **OR (S vs NS) (90% CI)** | **P value** |
| --- | --- | --- |
| Unadjusted (S vs NS) | 1.102 (0.981, 1.238) | 0.1680 |
| Adjusted for^a^: |  |  |
| Study Design^b^ | 1.036 (0.916, 1.173) | 0.6342 |
| Patient Population^c^ | 1.054 (0.928, 1.196) | 0.4975 |
| Duration on Study Drug before Switch (Days) | 1.061 (0.892, 1.263) | 0.5732 |
| STP Duration (Weeks) | 1.023 (0.904, 1.158) | 0.7615 |
| Number of switches during STP | 1.024 (0.906, 1.158) | 0.7505 |
| Mean Age | 0.967 (0.853, 1.096) | 0.6578 |
| Gender (%) | 1.015 (0.896, 1.150) | 0.8447 |
| Mean BMI | 1.011 (0.859, 1.190) | 0.9103 |

^a^Adjusted odds ratios are from multiple logistic regression models adjusted by each individual factor and the biosimilar drugs (i.e., BLA study number).

^b^Study Design: Randomized Double Blind, Randomized Open Label, Open Label Extension.

^c^Patient Population: Ankylosing Spondylitis, Breast Cancer, Crohn Disease, Chronic Kidney Disease, Follicular Lymphoma, Psoriatic Arthritis, Plaque Psoriasis, Rheumatoid Arthritis, Spondylarthritis, Type 1 Diabetes Mellitus, Ulcerative Colitis

**Table S12. Un-adjusted and adjusted odds ratio of discontinuation by individual factor and biosimilar drug.**

Exploratory Logistic regression analysis was performed to evaluate the impact of heterogeneity of different studies on study conclusions. Adjusted Odds Ratios (OR) from multiple logistic regression models were adjusted for the biosimilar drugs assessed and each individual factor (study design, patient population, time on study drug prior to switching, duration of switch, number of switches during STP, mean age, percent sex, and mean BMI).

| **Factor** | **OR (S vs NS) (90% CI)** | **P value** |
| --- | --- | --- |
| Unadjusted (S vs NS) | 0.974 (0.804, 1.181) | 0.8241 |
| Adjusted for^a^: |  |  |
| Study Design^b^ | 0.988 (0.814, 1.199) | 0.9164 |
| Patient Population^c^ | 0.981 (0.809, 1.191) | 0.8731 |
| Period 1 Duration (Days) | 1.081 (0.826, 1.416) | 0.6336 |
| Duration on Study Drug before Switch (Days) | 0.974 (0.803, 1.182) | 0.8228 |
| Number of switches during STP | 0.984 (0.811, 1.195) | 0.8928 |
| Mean Age | 0.955 (0.784, 1.163) | 0.7008 |
| Gender (%) | 0.982 (0.805, 1.197) | 0.8789 |
| Mean BMI | 1.043 (0.819, 1.329) | 0.7724 |

^a^Adjusted odds ratios are from multiple logistic regression models adjusted by each individual factor and the biosimilar drugs (i.e., BLA study number).

^b^Study Design: Randomized Double Blind, Randomized Open Label, Open Label Extension.

^c^Patient Population: Ankylosing Spondylitis, Breast Cancer, Crohn Disease, Chronic Kidney Disease, Follicular Lymphoma, Psoriatic Arthritis, Plaque Psoriasis, Rheumatoid Arthritis, Spondylarthritis, Type 1 Diabetes Mellitus, Ulcerative Colitis

References

1. U.S. Food and Drug Administration. Biosimilars Silver Spring, MD: U.S. Food and Drug Administration; 2022 [Available from: <https://www.fda.gov/drugs/therapeutic-biologics-applications-bla/biosimilars>.

2. U.S. Food and Drug Administration. Draft Guidance for Industry: Development of Therapeutic Protein Biosimilars: Comparative Analytical Assessment and Other Quality-Related Considerations Silver Spring, MD2019.

3. U.S. Food and Drug Administration. Guidance for Industry: Scientific considerations in demonstrating biosimilarity to a reference product. Silver Spring, MD. 2015.

4. Wells GA, Shea B, O’Connell D, Peterson J, Welch V, Losos M, et al. The Newcastle-Ottawa Scale (NOS) for assessing the quality of nonrandomised studies in meta-analyses. Oxford; 2000.

5. Douketis J, Tosetto A, Marcucci M, Baglin T, Cosmi B, Cushman M, et al. Risk of recurrence after venous thromboembolism in men and women: Patient level meta-analysis. BMJ. 2011;342(7796):535.

6. Khan F, Tritschler T, Kimpton M, Wells PS, Kearon C, Weitz JI, et al. Long-term risk for major bleeding during extended oral anticoagulant therapy for first unprovoked venous thromboembolism: A systematic review and meta-analysis. Annals of Internal Medicine. 2021;174(10):1420-9.

7. U.S. Food and Drug Administration. Drug approval package: adalimumab-aacf. Silver Spring, MD: U.S. Food and Drug Administration December 12, 2022. Contract No.: BLA 761255.

8. US Food and Drug Administration. Drug approval package: adalimumab-adaz. Silver Spring, MD: US Food and Drug Administration October 30, 2018. Contract No.: BLA 761071.

9. Wiland P, Jeka S, Dokoupilová E, Brandt-Jürgens J, Miranda Limón JM, Cantalejo Moreira M, et al. Switching to Biosimilar SDZ-ADL in Patients with Moderate-to-Severe Active Rheumatoid Arthritis: 48-Week Efficacy, Safety and Immunogenicity Results From the Phase III, Randomized, Double-Blind ADMYRA Study. BioDrugs. 2020;34(6):809-23.

10. U.S. Food and Drug Administration. Drug approval package: adalimumab-adbm. Silver Spring, MD: US Food and Drug Administration August 25, 2017. Contract No.: BLA 761058.

11. U.S. Food and Drug Administration. Drug approval package: adalimumab-afzb. Silver Spring, MD: US Food and Drug Administration November 15, 2019. Contract No.: BLA 761118.

12. Fleischmann RM, Alvarez DF, Bock AE, Cronenberger C, Vranic I, Zhang W, et al. Long-term efficacy, safety, and immunogenicity of the adalimumab biosimilar, PF-06410293, in patients with rheumatoid arthritis after switching from reference adalimumab (Humira®) or continuing biosimilar therapy: week 52–92 data from a randomized, double-blind, phase 3 trial. Arthritis Research and Therapy. 2021;23(1).

13. U.S. Food and Drug Administration. Drug approval package: adalimumab-aqvh. Silver Spring, MD: US Food and Drug Administration December 17, 2021. Contract No.: BLA 761216.

14. U.S. Food and Drug Administration. Drug approval package: adalimumab-atto. Silver Spring, MD: US Food and Drug Administration September 23, 2016. Contract No.: BLA 761024.

15. Papp K, Bachelez H, Costanzo A, Foley P, Gooderham M, Kaur P, et al. Clinical similarity of the biosimilar ABP 501 compared with adalimumab after single transition: long-term results from a randomized controlled, double-blind, 52-week, phase III trial in patients with moderate-to-severe plaque psoriasis. British Journal of Dermatology. 2017;177(6):1562-74.

16. Cohen S, Pablos JL, Pavelka K, Müller GA, Matsumoto A, Kivitz A, et al. An open-label extension study to demonstrate long-term safety and efficacy of ABP 501 in patients with rheumatoid arthritis. Arthritis Research and Therapy. 2019;21(1).

17. U.S. Food and Drug Administration. Drug approval package: adalimumab-bwwd. Silver Spring, MD: US Food and Drug Administration July 23, 2019. Contract No.: BLA 761059.

18. Weinblatt ME, Baranauskaite A, Dokoupilova E, Zielinska A, Jaworski J, Racewicz A, et al. Switching From Reference Adalimumab to SB5 (Adalimumab Biosimilar) in Patients With Rheumatoid Arthritis: Fifty-Two–Week Phase III Randomized Study Results. Arthritis and Rheumatology. 2018;70(6):832-40.

19. U.S. Food and Drug Administration. Drug approval package: adalimumab-fkjp. Silver Spring, MD: US Food and Drug Administration July 6, 2020. Contract No.: BLA 761154.

20. U.S. Food and Drug Administration. Drug approval package: epoetin alfa-epbx. Silver Spring, MD: US Food and Drug Administration May 15, 2018. Contract No.: BLA 125545.

21. Fishbane S, Singh B, Kumbhat S, Wisemandle WA, Martin NE. Intravenous epoetin alfa-epbx versus epoetin alfa for treatment of anemia in end-stage kidney disease. Clinical Journal of the American Society of Nephrology. 2018;13(8):1204-14.

22. Thadhani R, Guilatco R, Hymes J, Maddux FW, Ahuja A. Switching from epoetin alfa (Epogen®) to epoetin alfa-epbx (RetacritTM) using a specified dosing algorithm: A randomized, non-inferiority study in adults on hemodialysis. American Journal of Nephrology. 2018;48(3):214-24.

23. U.S. Food and Drug Administration. Drug approval package: etanercept-szzs. Silver Spring, MD: US Food and Drug Administration August 30, 2016. Contract No.: BLA 761042.

24. Jaworski J, Matucci-Cerinic M, Schulze-Koops H, Buch MH, Kucharz EJ, Allanore Y, et al. Switch from reference etanercept to SDZ ETN, an etanercept biosimilar, does not impact efficacy, safety, and immunogenicity of etanercept in patients with moderate-to-severe rheumatoid arthritis: 48-week results from the phase III, randomized, double-blind EQUIRA study. Arthritis Research and Therapy. 2019;21(1).

25. U.S. Food and Drug Administration. Drug approval package: etanercept-ykro. Silver Spring, MD: US Food and Drug Administration April 25, 2019. Contract No.: BLA 761066.

26. Emery P, Vencovský J, Sylwestrzak A, Leszczyński P, Porawska W, Stasiuk B, et al. Long-term efficacy and safety in patients with rheumatoid arthritis continuing on SB4 or switching from reference etanercept to SB4. Annals of the Rheumatic Diseases. 2017;76(12):1986-91.

27. Blackwell K, Gascon P, Krendyukov A, Gattu S, Li Y, Harbeck N. Safety and efficacy of alternating treatment with EP2006, a filgrastim biosimilar, and reference filgrastim: A phase III, randomised, double-blind clinical study in the prevention of severe neutropenia in patients with breast cancer receiving myelosuppressive chemotherapy. Annals of Oncology. 2018;29(1):244-9.

28. U.S. Food and Drug Administration. Drug approval package: infliximab-abda. Silver Spring, MD: US Food and Drug Administration April 21, 2017. Contract No.: BLA 761054.

29. U.S. Food and Drug Administration. Drug approval package: infliximab-axxq. Silver Spring, MD: US Food and Drug Administration December 6, 2019. Contract No.: BLA 761086.

30. U.S. Food and Drug Administration. Drug approval package: infliximab-dyyb. Silver Spring, MD: US Food and Drug Administration April 5, 2016. Contract No.: BLA 125544.

31. Yoo DH, Prodanovic N, Jaworski J, Miranda P, Ramiterre E, Lanzon A, et al. Efficacy and safety of CT-P13 (biosimilar infliximab) in patients with rheumatoid arthritis: Comparison between switching from reference infliximab to CT-P13 and continuing CT-P13 in the PLANETRA extension study. Annals of the Rheumatic Diseases. 2017;76(2):355-63.

32. Park W, Yoo DH, Miranda P, Brzosko M, Wiland P, Gutierrez-Ureña S, et al. Efficacy and safety of switching from reference infliximab to CT-P13 compared with maintenance of CT-P13 in ankylosing spondylitis: 102-week data from the PLANETAS extension study. Annals of the Rheumatic Diseases. 2017;76(2):346-54.

33. Jørgensen KK, Olsen IC, Goll GL, Lorentzen M, Bolstad N, Haavardsholm EA, et al. Switching from originator infliximab to biosimilar CT-P13 compared with maintained treatment with originator infliximab (NOR-SWITCH): a 52-week, randomised, double-blind, non-inferiority trial. The Lancet. 2017;389(10086):2304-16.

34. Goll GL, Jørgensen KK, Sexton J, Olsen IC, Bolstad N, Haavardsholm EA, et al. Long-term efficacy and safety of biosimilar infliximab (CT-P13) after switching from originator infliximab: open-label extension of the NOR-SWITCH trial. Journal of Internal Medicine. 2019;285(6):653-69.

35. Ye BD, Pesegova M, Alexeeva O, Osipenko M, Lahat A, Dorofeyev A, et al. Efficacy and safety of biosimilar CT-P13 compared with originator infliximab in patients with active Crohn's disease: an international, randomised, double-blind, phase 3 non-inferiority study. The Lancet. 2019;393(10182):1699-707.

36. U.S. Food and Drug Administration. Drug approval package: infliximab-qbtx. Silver Spring, MD: US Food and Drug Administration December 13, 2017. Contract No.: BLA 761072.

37. Cohen SB, Radominski SC, Kameda H, Kivitz AJ, Tee M, Cronenberger C, et al. Long-term Efficacy, Safety, and Immunogenicity of the Infliximab (IFX) Biosimilar, PF-06438179/GP1111, in Patients with Rheumatoid Arthritis After Switching from Reference IFX or Continuing Biosimilar Therapy: Week 54–78 Data From a Randomized, Double-Blind, Phase III Trial. BioDrugs. 2020;34(2):197-207.

38. U.S. Food and Drug Administration. Drug approval package: insulin glargine-yfgn. Silver Spring, MD: US Food and Drug Administration July 28, 2021. Contract No.: BLA 761201.

39. U.S. Food and Drug Administration. Drug approval package: rituximab-abbs. Silver Spring, MD: US Food and Drug Administration November 28, 2018. Contract No.: BLA 761088.

40. Shim SC, Božić-Majstorović L, Berrocal Kasay A, El-Khouri EC, Irazoque-Palazuelos F, Cons Molina FF, et al. Efficacy and safety of switching from rituximab to biosimilar CT-P10 in rheumatoid arthritis: 72-week data from a randomized Phase 3 trial. Rheumatology (United Kingdom). 2019;58(12):2193-202.

41. Kwak LW, Sancho JM, Cho SG, Nakazawa H, Suzumiya J, Tumyan G, et al. Efficacy and Safety of CT-P10 Versus Rituximab in Untreated Low-Tumor-Burden Follicular Lymphoma: Final Results of a Randomized Phase III Study. Clinical Lymphoma, Myeloma and Leukemia. 2022;22(2):89-97.

42. Ogura M, Sancho JM, Cho SG, Nakazawa H, Suzumiya J, Tumyan G, et al. Efficacy, pharmacokinetics, and safety of the biosimilar CT-P10 in comparison with rituximab in patients with previously untreated low-tumour-burden follicular lymphoma: a randomised, double-blind, parallel-group, phase 3 trial. Lancet Haematology. 2018;5(11):e543-e53.

43. U.S. Food and Drug Administration. Drug approval package: rituximab-arrx. Silver Spring, MD: US Food and Drug administration December 17, 2020. Contract No.: BLA 761140.

44. U.S. Food and Drug Administration. Drug approval package: rituximab-pvvr. Silver Spring, MD: US Food and Drug Administration July 23, 2019. Contract No.: BLA 761103.

45. Cohen SB, Burgos-Vargas R, Emery P, Jin B, Cronenberger C, Vázquez-Abad MD. Extension Study of PF-05280586, a Potential Rituximab Biosimilar, Versus Rituximab in Subjects With Active Rheumatoid Arthritis. Arthritis Care and Research. 2018;70(11):1598-606.

46. U.S. Food and Drug Administration. Drug approval package: trastuzumab-anns. Silver Spring, MD: US Food and Drug administration June 13, 2019. Contract No.: BLA 761073.

47. von Minckwitz G, Colleoni M, Kolberg HC, Morales S, Santi P, Tomasevic Z, et al. Efficacy and safety of ABP 980 compared with reference trastuzumab in women with HER2-positive early breast cancer (LILAC study): a randomised, double-blind, phase 3 trial. The Lancet Oncology. 2018;19(7):987-98.
